# Small molecule valproic acid enhances ventral patterning of human neural tube organoids by regulating Wnt and Shh signalling

**DOI:** 10.1111/cpr.13737

**Published:** 2024-08-20

**Authors:** Yuanyuan Zheng, Fangrong Zhang, Haifeng Nie, Xinyu Li, Jiali Xun, Jianping Fu, Lijun Wu

**Affiliations:** ^1^ Information Materials and Intelligent Sensing Laboratory of Anhui Province, Institute of Physical Science and Information Technology Anhui University Hefei China; ^2^ Department of Mechanical Engineering University of Michigan Ann Arbor Michigan USA; ^3^ Department of Cell & Developmental Biology University of Michigan Medical School Ann Arbor Michigan USA; ^4^ Department of Biomedical Engineering University of Michigan Ann Arbor Michigan USA

## Abstract

Valproic acid (VPA), a clinically approved small molecule, has been reported to activate Wnt signalling that is critical for dorsal–ventral (DV) patterning of neural tube. However, little is known about the impact of VPA on DV patterning process. Here, we show that even though VPA has a negative impact on the early formation of human neural tube organoids (hNTOs), it significantly enhances the efficiency of ventrally patterned hNTOs, when VPA is added during the entire differentiation process. RNA sequencing and RT‐qPCR analysis demonstrates VPA activates endogenous Wnt signalling in hNTOs. Surprisingly, transcriptome analysis also identifies upregulation of genes for degradation of GLI2 and GLI3 proteins, whose truncated fragment are transcriptional repressors of Shh signalling. The Western‐blot analysis confirms the increase of GLI3R proteins after VPA treatment. Thus, VPA might enhance ventral patterning of hNTOs through both activating Wnt, which can antagonise Shh signalling by inducing GLI3 expression, and/or inhibiting Shh signalling by inducing GLI protein degradation. We further obtain results to show that VPA still increases patterning efficiency of hNTOs with a weak influence on their early formation when the initiation time of VPA is delayed and its duration is reduced. Taken together, this study demonstrates that VPA enhances the generation of more reproducible hNTOs with ventral patterning, opening the avenues for the applications of hNTOs in developmental biology and regenerative medicine.

## Graphical abstract

This study shows that small molecule valproic acid enable the generation of more reproducible, ventrally self‐patterned hNTOs, in which floor plate and pMN domains were spatially patterned at one pole of the organoid, by inducing Wnt signalling and suppressing Shh signalling.

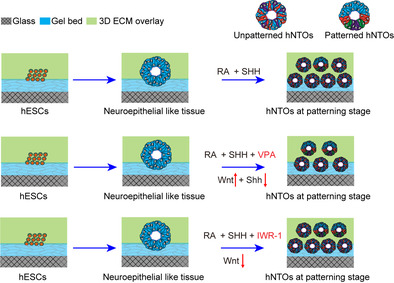

## 1 INTRODUCTION

The formation of neural tube begins with the rolling up of the sheet‐like neural plate, which then closes at the dorsal midline of the embryo to form a tubular structure, the neural tube.^1^ Following neural tube formation, the progenitor cells in the neural tube, or neuroepithelial cells, give rise to distinct neuronal progenitor cells and subsequently distinct cell types at precise locations along the dorsoventral (DV) axis of the developing embryo.^2^ DV patterning of the neural tube is thought to be controlled by the actions of multiple factors.^3^ Bone morphogenetic proteins (BMPs) and wingless‐type MMTV integration site protein family (WNT), produced by most dorsally located cells of the neural tube (the roof plate), induces the generation of dorsal progenitor domains in the neural tube.^4^, ^5^, ^6^ Concurrently, sonic hedgehog (SHH) produced by most ventrally located cells of the neural tube (the floor plate) induces ventral patterning of the neural tube.^7^

Valproic acid (VPA), a clinically approved small molecule, was recently reported to activate Wnt signalling. For example, VPA has been shown to effectively stimulate hair follicle regrowth by upregulating Wnt/β‐catenin and thus can be used as a potential candidate for inducing hair regrowth.^8^ In particularly, Wang and colleagues report that VPA could induce neuronal differentiation of neural stem cells (NSCs) by activating Wnt signalling.^9^ Consistently, Park and co‐workers show that Wnt/β‐catenin signalling is activated in VPA‐exposed mouse model of autism spectrum disorder.^10^ Importantly, exposing human cortical organoids to VPA decreased expression of *Lhx9*, which negatively regulates Wnt signalling.^11^, ^12^ Together, these findings support a role of VPA in upregulating Wnt signalling, suggesting that VPA might have the ability to alter DV patterning of the neural tube through activating Wnt signalling.

Recently, with advanced technologies of organoid culturing, patterned human neural tube organoids (hNTOs) have been generated from human pluripotent stem cells (hPSCs).^13^, ^14^ The hNTOs with spatially specialised neurons is promising for the regeneration of spinal‐cord injury, disease modelling and developmental biology applications.^15^, ^16^ The current protocols for generating DV patterned hNTOs rely on retinoic acid (RA) and/or SHH without controlling exogeneous Wnt signalling. Thus, the hNTO model provides a valuable experimental tool to investigate the impact of VPA on DV patterning of the neural tube. VPA has been developed to generate induced pluripotent stem cells with preservation of genomic integrity, and even to repair spinal cord injury.^17^, ^18^ Therefore, considering the role of Wnt signalling in dorsal patterning of the neural tube and that VPA could activate Wnt signalling, we hypothesise that modulation of VPA in the hNTOs culture will promote dorsal differentiation of hNTOs.

In this study, we present the first data to explore the impact of VPA on DV patterning of neural tube based on hNTOs modified from our previous study.^13^ We first investigated the effects of VPA below therapeutic concentrations (350–700 μM in serum)^19^ on hNTOs early formation and their subsequent DV patterning. We then applied both RNA‐seq and RT‐qPCR to examine gene expression pattern changes in hNTOs as a function of VPA treatment to confirm whether Wnt signalling was perturbed by VPA treatment. We further explored ways in which VPA could alter DV patterning of hNTOs but without affecting their early formation. Together, our study developed a reliable strategy to modulate DV patterning of hNTOs and thus has the penitential for expanding applications of hNTOs in developmental biology, regenerative medicine and drug screening.

## 2 RESULTS

### 2.1 Construction of DV patterned hNTOs in a biomimetic 3D culture

**FIGURE 1:**
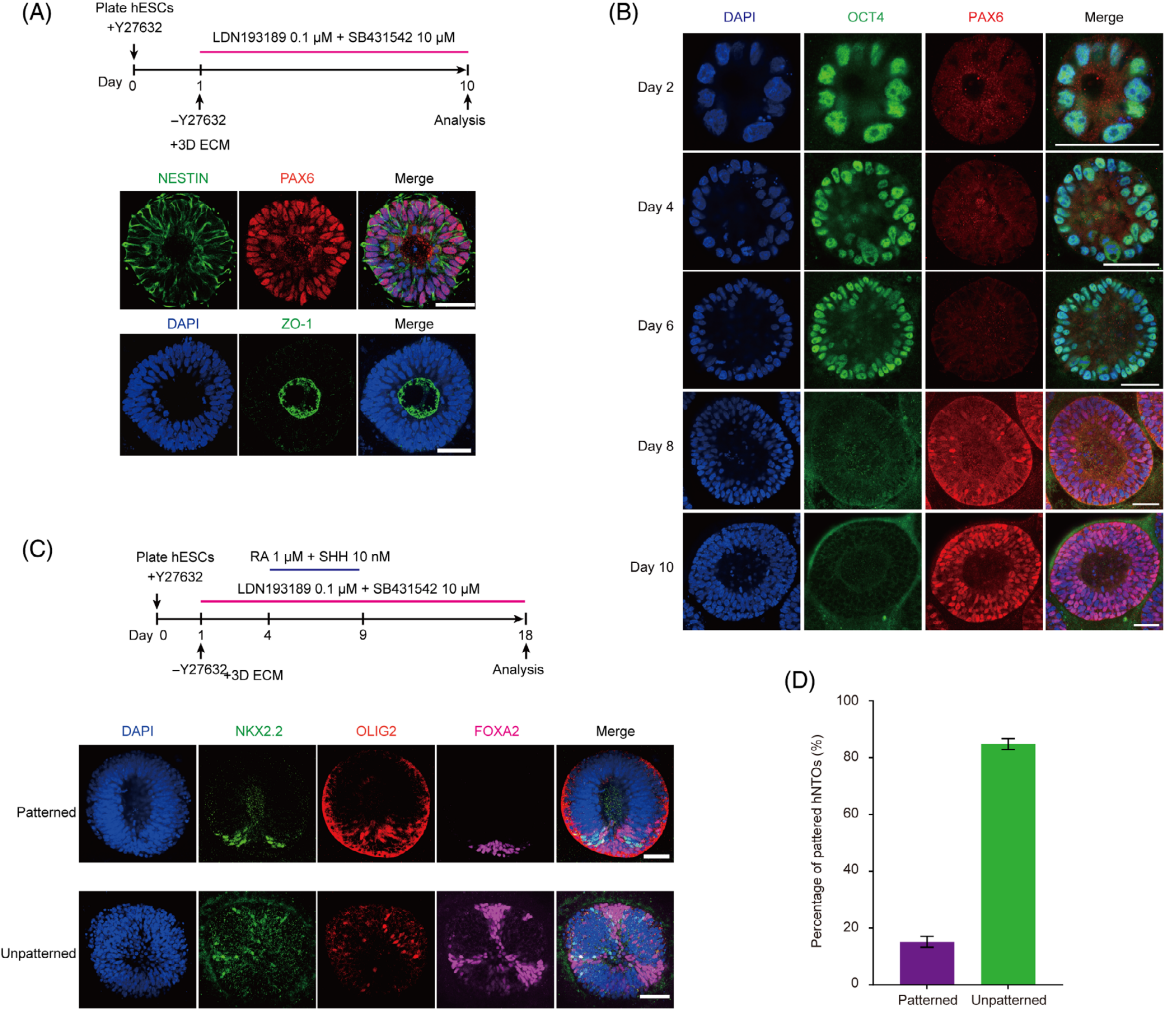
The generation of dorsal–ventral (DV) patterned neural tube organoid. (A) Representative confocal micrographs of organoids at Day 10 exhibiting multicellular pseudostratified neuroepithelial tissue with lumen. DAPI‐counterstained nuclei. (B) Confocal micrographs showing the exit of pluripotency and neural conversion of hESCs in organoids. DAPI counterstained nuclei. (C) Representative confocal micrographs showing ventral patterned organoids obtained at day 18 stained for FOXA2, OLIG2, and NKX2.2 as indicated. DAPI counterstained nuclei. (D) Histogram graph showing percentages of 2 types organoids at days 18. A total of 200–400 cysts were counted from *n* = 4 independent experiments at day 18. Error bars are SEM. Scale bars, 50 μm (A, B and C).

To understand the impact of VPA on neural tube patterning, we first established a patterned hNTOs model from human embryonic stem cells (hESCs) in a biomimetic 3D culture based on our previous study.^13^ Specifically, hESCs were seeded as single cells at a density of 30,000 cells cm^−2^ onto a thick, soft gel bed of Geltrex in mTeSR1 medium supplemented with Y27632. On the following day (day 1), N2SM1 medium supplemented with 2% (v/v) Geltrex was added to establish a 3D extracellular environment. At the same time, BMP inhibitor LDN193189 (LDN) and TGF‐β signalling inhibitor SB431542 (SB) were added to N2SM1 medium to induce neural differentiation of hESCs (Figure 1A). Under this neural induction condition for 10 days, pseudostratified, neuroepithelial (NE)‐like tissues containing a central lumen were generated. Immunostaining analysis on day 10 revealed that these NE‐like tissues express neuroepithelial markers PAX6 and NESTIN (Figure 1A). Furthermore, apical localization of the tight junction marker ZO‐1 was evident for these NE‐like tissues, supporting that the apicobasal polarity was established in these organoids (Figure 1A).

To examine the dynamics of cell fate specialisation in the NE‐like tissues, we further conducted immunostaining to stain for the early NE marker PAX6^20^ and pluripotency marker OCT4.^21^ The results showed that cells in organoids strongly expressed OCT4 but not PAX6 from day 0 to day 6 (Figure 1B). PAX6+ cells were detected on Day 8. By day 10, most of organoids contained only PAX6+ OCT4‐NE cells, suggesting completion of neural conversion, consistent with our previous results.^13^

To investigate whether VPA affect DV patterning of neural tube through modulating Wnt signalling, we examined the generation of ventrally patterned hNTOs. Specifically, 10 nM SHH combined with 1 μM RA were supplemented in N2SM1 medium between day 4 and day 9 of differentiation for ventral patterning of hNTOs. Neural tube ventral‐most floor plate (FP) domain marker FOXA2, ventral V3 domain maker NKX2.2 and ventral pMN domain maker OLIG2 were examined to assess patterning of hNTOs. With RA and 10 nM SHH, ventral patterning of hNTOs with properly aligned pMN, p3, and FP domains was achieved (Figure 1C). The results show that in OLIG2+ FOXA2+ hNTOs, 15% of them displayed proper OLIG2+ FOXA2+ patterning on day 18 (Figure 1D). We also observed PAX3+ FOXA2+ patterning in one hNTO (Figure S1); however, the number of such patterned organoids was particularly small. Therefore, in subsequent experiments, we focused on the ventrally patterned organoids.

### 2.2 VPA inhibited formation of early hNTOs

To investigate the effect of VPA treatment on early development of hNTOs, VPA was added during the entire neural induction process of hNTO formation. Cell viability assay was conducted, revealing that VPA treatment resulted in significant decrease of cell viability with VPA concentration above 300 μM (Figure S2). We should note here that the therapeutic range for total serum VPA is 350 to 700 μM in humans, suggesting that such high concentrations of VPA used in clinical settings might have a negative impact of neural tube development.^22^

**FIGURE 2:**
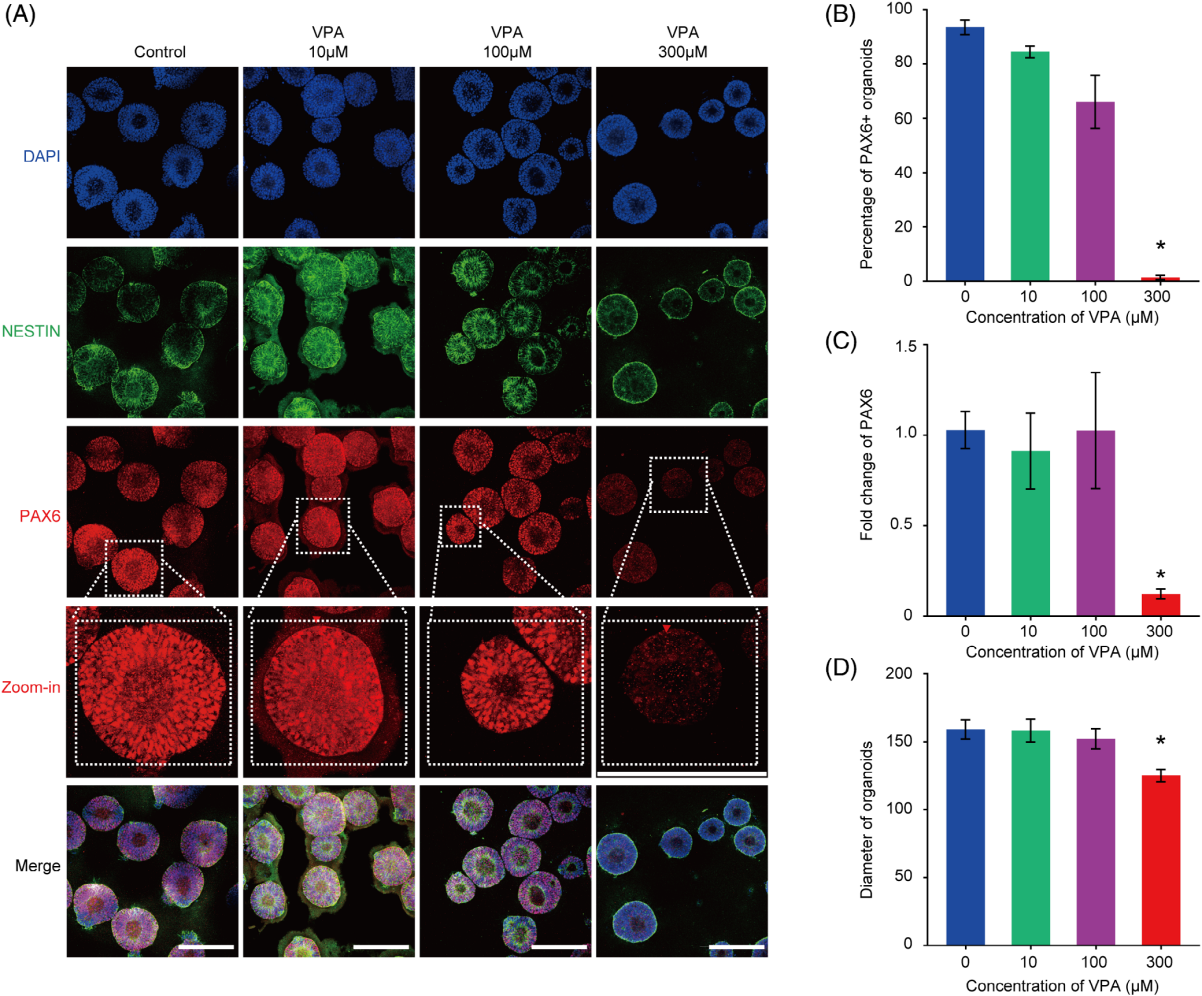
Valproic acid (VPA) treatment inhibited the formation of primary hNTOs. (A) Representative image of 0, 10, 100, and 300 μM VPA‐treated primary hNTOs for PAX6 (red) and NESTIN (green) at day 10. The zoomed‐in images showing a magnified view of the area highlighted by the white square. Scale bars, 100 μm. (B) Quantification of the ratios of PAX6+ organoids at day 10 (*n* > 200 organoids each experiment). (C) RT‐qPCR analysis displaying relative mRNA expression of marker gene PAX6 of 0, 10, 100, and 300 μM VPA‐treated organoids. Experiments were performed in three biological replicates. (D) Primary hNTOs treated with 300 μM VPA showing a decreased size. Data bars in B, C and D represent mean ± SEM. *p ≤ 0.05.

Thus, in our following experiments, we chose to use 300 μM VPA or even lower doses of VPA to treat hNTOs to explore the effect of VPA on hNTO development at different stages. Formation of hNTOs was analysed on day 10, and NE marker PAX6 and NESTIN were used for immunofluorescence staining (Figure 2A). The number of PAX6+ cells in VPA‐treated hNTOs was clearly decreased (Figure 2A). The effect of VPA on NESTIN expression was not as obvious. It may be that NESTIN is expressed on the cell membrane, thus different expression levels of NESTIN might not be easy to distinguish in immunostaining assays. Therefore, we further quantified the efficiency of PAX6+ hNTOs under different VPA dose conditions. Consistent with the immunofluorescence results, 300 μM VPA treatment decreased significantly the efficiency of hNTO formation. Lower doses of VPA had no significant effect on the efficiency of PAX6+ hNTO formation (Figure 2B). Based on RT‐qPCR measurements of *PAX6*, we observed that treatment with 300 μM VPA inhibited *PAX6* expression, supporting that VPA reduced NE differentiation at this dose (Figure 2C). In addition, we found that exposure to VPA could also affect hNTO growth. The diameter of hNTOs was indistinguishable in hNTOs when VPA dose was below 100 μM compared with untreated controls. However, hNTOs treated with 300 μM VPA displayed markedly smaller diameter than control organoids (Figure 2D). These results suggest that clinically relevant doses of VPA could inhibit the development of NEs in hNTOs.

### 2.3 VPA enhanced ventral patterning efficiency of hNTOs

**FIGURE 3:**
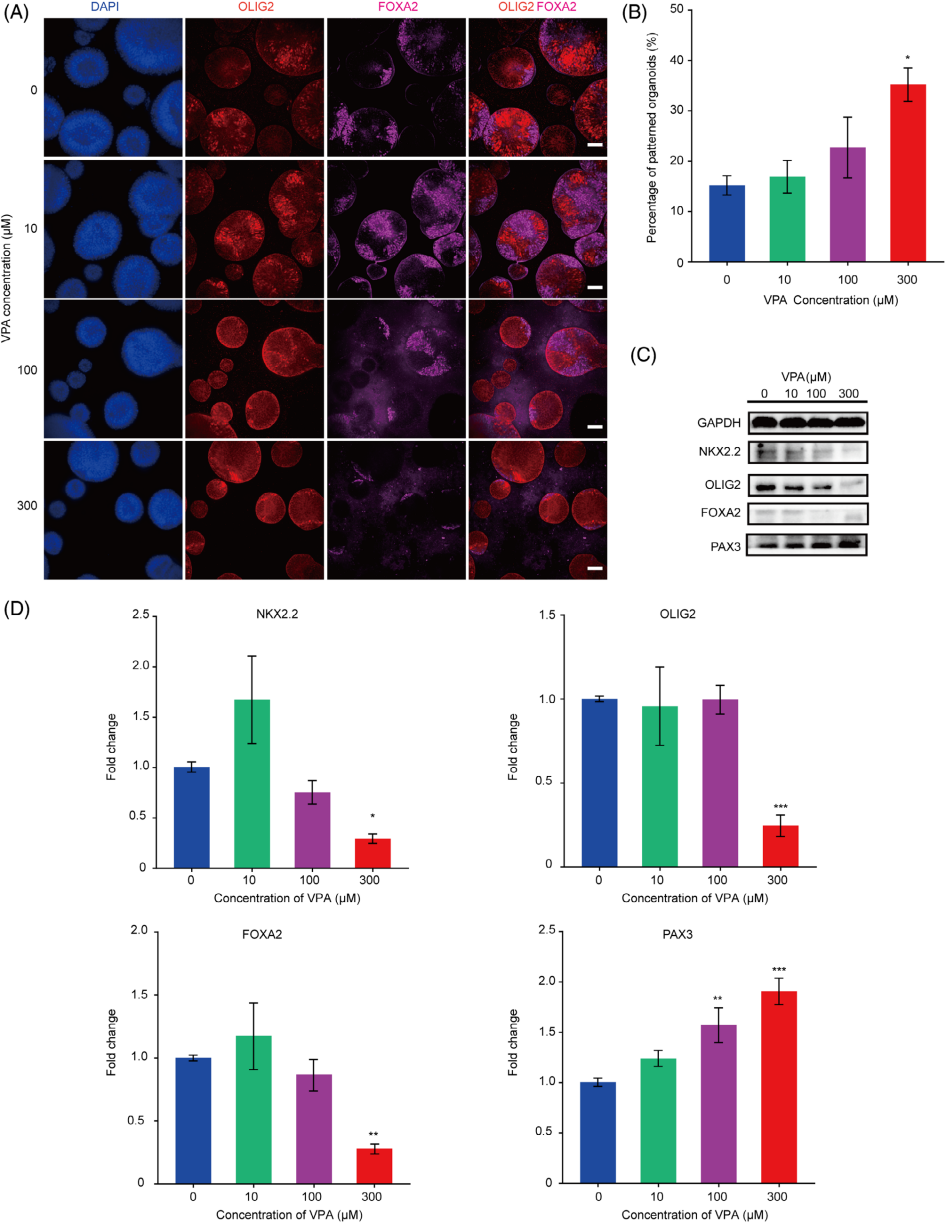
Valproic acid (VPA) treatment enhanced the ventral patterning efficiency of hNTOs. (A) Representative image of 0, 10, 100, and 300 μM VPA‐treated hNTOs for OLIG2 (red) and FOXA2 (magenta) at day 18. Scale bars, 50 μm. (B) Quantification of the percentages of OLIG2 + FOXA2+ patterned organoids at day 18 (300 organoids were counted for each condition in one experiment). *n* = 3 independent experiments. Data bars represent mean ± SEM. *p ≤ 0.05. (C) Sample images of Western‐blot analysis of ventral marker NKX2.2, OLIG2, FOXA2 and dorsal marker PAX3 protein levels in organoids treated by different concentration VPA. (D) RT‐qPCR analysis displaying relative mRNA expression of genes *NKX2.2*, *OLIG2*, *FOXA2* and *PAX3* of 0, 10, 100, and 300 μM VPA‐treated organoids. Experiments were performed in three biological replicates. *p ≤ 0.05, **p ≤ 0.01, ***p ≤ 0.001.

To investigate the impact of VPA on early human neural fate specification and patterning, we detected expression of neural tube ventral markers OLIG2 and FOXA2 by immunostaining. Immunofluorescence images recorded on day 18 revealed that the numbers of OLIG2+ and FOXA2+ cells in hNTOs were decreased under 300 μM VPA treatment. Intriguingly, the number of hNTOs exhibiting patterned OLIG2+ and FOXA2+ domains was notably increased (Figure 3A). Therefore, we next quantified the efficiency of ventral patterning of hNTOs, in terms of patterned OLIG2+ and FOXA2+ domains, under different conditions. On day 18, 15% of hNTOs in control group achieved ventral patterning, with two OLIG2+ pMN domains positioned dorsal to a single FOXA2+ FP domain (Figure 3A,B). Strikingly, VPA enhanced the efficiency of hNTOs with patterned OLIG2+ and FOXA2+ domains, from 15% in control groups to a maximum of 41% under 300 μM VPA treatments (Figure 3B).

We further quantified expression levels of ventral neural tube markers NKX2.2, OLIG2, FOXA2 as well as dorsal neural tube marker PAX3. We treated hNTOs with different doses of VPA or vehicle controls and harvested cell lysates on day 18. The cell lysates were analysed by Western‐blot using antibodies specific for NKX2.2, OLIG2, FOXA2 and PAX3. Consistent with the results of immunofluorescence, levels of NKX2.2, OLIG2 and FOXA2 substantially decreased in hNTOs treated with VPA, especially for those treated with 300 μM VPA (Figure 3C). In contrast, PAX3 expression increased in hNTOs treated with VPA. To validate our observations, we also performed RT‐qPCR analysis for the expression change of above marker genes. Consistently, hNTOs cultured under 300 μM VPA expressed lower levels of mRNA transcripts of *NKX2.2*, *OLIG2*, and *FOXA2* but a greater level of *PAX3* than untreated controls (Figure 3D). Taken together, the above results suggest that VPA treatment affected neural fate specification and ventral patterning of hNTOs.

### 2.4 VPA upregulated Wnt signalling at patterning stage

**FIGURE 4:**
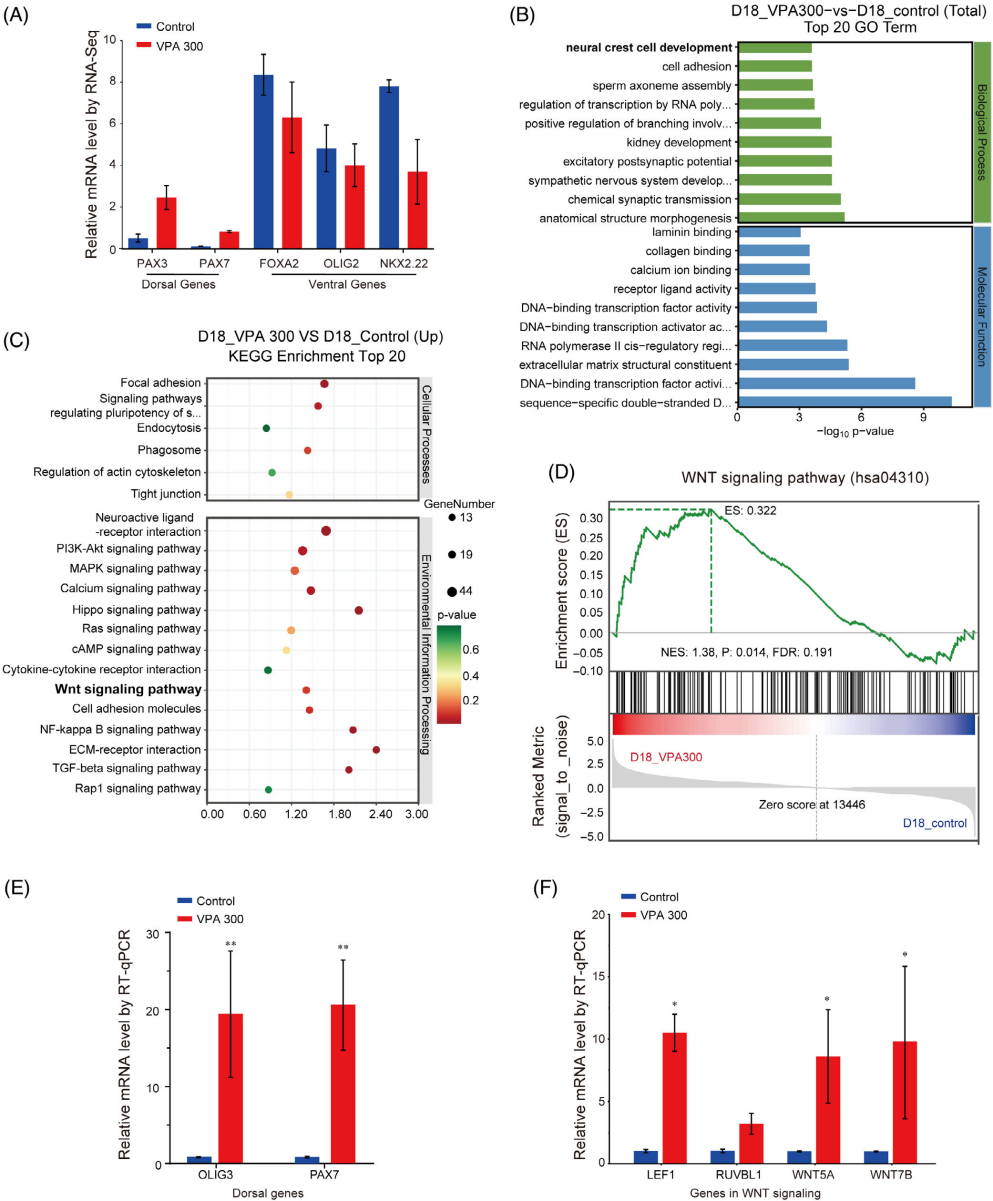
Wnt signalling was upregulated in VPA‐treated hNTOs compared with control hNTOs. (A) The graph shows RNA‐seq results (normalised FPKM) of dorsal and ventral genes. The normalised FPKM of dorsal genes were increased, whereas the mRNA expression of ventral genes was decreased in VPA‐treated hNTOs. (B) The bar graph shows the top 10 significant items in the biological process and molecular function fractions based on the *p* values in the GO analysis. (C) KEGG enrichment analysis of up‐regulated DEGs revealed upregulated DEGs were mainly associated with Wnt, TGF‐β signalling, PI3K‐Akt signalling, Hippo signalling neuroactive ligand–receptor interaction, ECM–receptor interaction pathways in VPA‐treated hNTOs. (D) Gene set enrichment plot depicting the elevated positive regulation of the Wnt signalling pathway in VPA‐treated hNTOs. (E) The graph shows RT‐qPCR confirmation results for dorsal genes *PAX7* and *OLIG3* induced by Wnt signalling. (F) The graph shows RT‐qPCR confirmation results for selected genes enriched in Wnt signalling. The mRNA expression of *LEF1*, *RUVBL1*, *WNT5A* and *WNT7B* was increased in VPA‐treated hNTOs using RNA samples for RNA‐seq under independent triplicates per sample. Data bars in (E and F) represent mean ± SEM from three independent experiments. *p ≤ 0.05, **p < 0.01.

The above results suggest that VPA could impact DV patterning of hNTOs. Thus, we speculated that VPA might regulate signalling events that play an important role in DV patterning of the neural tube, including Wnt signalling. To investigate whether VPA induces transcriptional changes in hNTOs through Wnt signalling activation and to elucidate the molecular mechanism underlying the changes in ventral patterning induced by VPA, we performed RNA‐sequencing (RNA‐seq). Ventrally patterned hNTOs on day 18 were processed for RNA‐seq. Principal component analysis (PCA) and sample clustering were applied to determine the variance across different experimental groups and replicates. The PCA analysis results showed that VPA‐treated hNTOs and controls cluster into two distinct groups (Figure S3). PCA and hierarchical sample clustering analysis confirmed that the replicate data sets in different samples were highly correlated, demonstrating high reproducibility (SI Figure S2B). We identified genes with *p* value <0.05 and |log_2_ Fold Change| >1 as significant differentially expressed genes (DEGs). DEG analysis revealed significant upregulation of 1682 and downregulation of 1376 genes in VPA‐treated organoids when compared with untreated controls (Figure S4). Notably, volcano plot identified OLIG3 as one of the most strongly upregulated genes in VPA‐treated hNTOs. OLIG3 is a transcription factor expressed in progenitors that generate dI1–dI3 neurons in dorsal neural tube and importantly, its expression is regulated by Wnt signals.^23^ DEG analyses further revealed dorsal progenitor genes, such as *PAX3* and *PAX7*, were significantly upregulated in VPA‐treated hNTOs (Figure 4A). PAX3 is among the first markers that upregulate and delineate the dorsal neural tube and then together with PAX7 to specify interneurons within the dorsal neural tube.^24^ Similar with OLIG3, Wnt signalling mediated induction of PAX3 and PAX7.^25^ These results indicated that dorsal genes induced by Wnt signalling were upregulated in VPA‐treated hNTOs. In addition, consistent with RT‐qPCR results in Figure 3, DEG analyses identified downregulation of ventral progenitor genes F*OXA2*, *NKX2.2* and *OLIG2* (Figure 4A). Studies have provided evidence that canonical Wnt signalling is active in ventral neural tube at the time when ventral cell types are specified and that inhibition of Wnt signalling results in the expansion of ventrally located progenitors.^26^, ^27^ Collectively, the above results suggested that Wnt signalling, which has opposite effects on ventral cell fate specification with Shh signalling, might be upregulated in VPA‐treated hNTOs.

To better understand the function of upregulated DEGs in VPA‐treated hNTOs, we subjected the 1682 up‐regulated DEGs to GO analysis and KEGG analyses.^28^, ^29^ Figure 4B shows the top 15 significant GO terms for the 1682 upregulated DEGs in the Biological Process categories. GO annotation and significance analysis revealed that these DEGs were mainly involved in neural crest cell development. The top 20 significant KEGG pathways for the 1682 upregulated DEGs are shown in Figure 4C. As expected, KEGG pathway analyses revealed that these upregulated DEGs were mainly involved in Wnt signalling pathway. Upregulated TGF‐β signalling pathway was also identified by KEGG analysis in VPA‐treated hNTOs. However, in the differentiation medium, TGF‐β inhibitor SB431524 was added through the entire differentiation process to induce neural differentiation. Given that TGF‐β signalling pathway is one of the main signal transduction pathways that control cell proliferation and survival,^30^ we speculate that TGF‐β signalling pathway was mainly involved in cell proliferation and survival in the present study. Additionally, KEGG pathway analysis revealed that upregulated DEGs in VPA‐treated hNTOs were also associated with pathways including PI3K‐Akt signalling, Hippo signalling, neuroactive ligand–receptor interaction and ECM–receptor interaction pathways (Figure 4C). KEGG analysis conducted for the down‐regulated DEGs in VPA‐treated hNTOs shows a similar result (Figure S5). The PI3K/Akt signalling pathway plays a critical role in controlling cell survival, promoting proliferation and inhibiting apoptosis.^31^ The Hippo pathway is a signalling pathway that inhibits cell proliferation, and loss of Hippo signalling leads to organ overgrowth.^32^ Moreover, Hippo pathway was reported to crosstalk with PI3K‐Akt signalling to control cell proliferation and tissue growth.^33^ Therefore, enriched genes associated with PI3K‐Akt and Hippo signalling suggest that VPA induced toxicity to cells in hNTOs and those down‐regulated were involved in controlling proliferation and apoptosis. Besides, the enrichment of neuroactive ligand–receptor interaction and ECM–receptor interaction pathways suggested VPA treatments resulted in altered expression of genes encoding proteins that perform functions in ligand–receptor interaction.

We also used gene set enrichment analysis (GSEA) as a bioinformatics tool.^34^ Enrichment for all sets of genes from the KEGG term database was performed by running GSEA against the test statistic‐ranked list of genes in the experiment. Notably, VPA treatment resulted in a significant enrichment with positive regulation of Wnt signalling pathways (KEGG: hsa04310; normalised enrichment score (NES) = 1.38, *p* = 0.014, FDR = 0.191) (Figure 4D). Importantly, Wnt signalling plays an essential and direct role in dorsal patterning of the neural tube and the induction of neural crest cells.^35^, ^36^ For example, transcription factors PAX3/7 induced by Wnt signalling play numerous roles in the development of the dorsal nervous system of vertebrates including specifying neural crest and patterning of dorsal neural tube.^37^ RT‐qPCR analysis confirmed upregulation of transcription factors *PAX3*, *PAX7* and *OLIG3* (Figures 3D and 4E). Thus, our RNA‐seq and RT‐qPCR data support that VPA likely regulates patterning of hNTOs through activating Wnt signalling pathway.

Specifically, RNA‐seq identified upregulation of 15 mRNAs enriched in Wnt signalling, including *APC2*, *BTRC*, *FRZB*, *FZD8*, *LEF1*, *LGR5*, *PRKACB*, *PRKCB*, *RUVBL1*, *SFRP2*, *SFRP5*, *TCF7*, *WNT11*, *WNT5A* and *WNT7B* (Figure S6). To further validate RNA‐seq results, we characterised the expression of some key genes in WNT signalling by RT‐qPCR analysis. As expected, VPA‐treated hNTOs showed greater expression of key genes, such as *LEF1*, *RUVBL1*, *WNT5A*, and *WNT7B*, than control ones (*p* < 0.05, Figure 4F). Among the genes involved in the WNT pathway, *WNT‐3A* is required for the specification of dorsal interneurons. *WNT7B* is a WNT ligand that has been demonstrated to activate canonical Wnt signalling.^38^ Elevated expression of *RUVBL1* is associated with poor survival of cells.^39^ *LEF1* has been reported to be directly activated by Wnt signalling, and its activated expression could activate the Wnt signalling pathway.^40^, ^41^ Therefore, the induction of *LEF1* by VPA treatment in hNTOs may be caused by activated Wnt signalling. As β‐catenin nuclear translocation is an important process in Wnt/β‐catenin signalling, we further investigated the effect of VPA on β‐catenin nuclear translocation. Western‐blot analysis of nuclear β‐catenin levels indicates that β‐catenin translocates into the nucleus in VPA‐treated groups (Figure S7A). However, β‐catenin protein levels in both nuclear and cytoplasmatic fractions were found to be unaffected by VPA treatment. Consistent with results of Western‐blot, the mRNA level of β‐catenin was not significantly affected after treatment with VPA (Figure S7B). Given that VPA increased the levels of downstream target genes *LEF1* and *TCF7*, these data suggest that VPA activated Wnt/β‐catenin signalling most possibly by activating downstream of β‐catenin.

Studies suggest that Wnt signalling directs the caudalization of neural plate cells during early neural tube development.^42^, ^43^ Thus, changes in Wnt signalling will lead to alteration of neural tube patterning along the anterior–posterior (AP) axis. To examine the role of VPA on AP patterning of hNOTs, we investigated the expression levels' change of HOX (A–D) genes upon VPA treatment using RNA‐seq data. The results show that RA induced highly expression of HOXA1‐HOXD4 genes, consistent with the role of RA in regulating colinear HOX1‐5 gene expression (Figure S8). Furthermore, we identified a caudalised shift of HOX genes from HOXB2 to HOXB4 after VPA treatment. Especially, the expression of HOXA4, HOXA5, HOXB1 and HOXD1 was significantly increased by VPA treatment. These results indicate that VPA could induce some degree of posterior identity, indirectly reflecting the activation of Wnt signalling after VPA treatment.

### 2.5 Degradation of GLI proteins was induced by VPA in hNTOs

**FIGURE 5:**
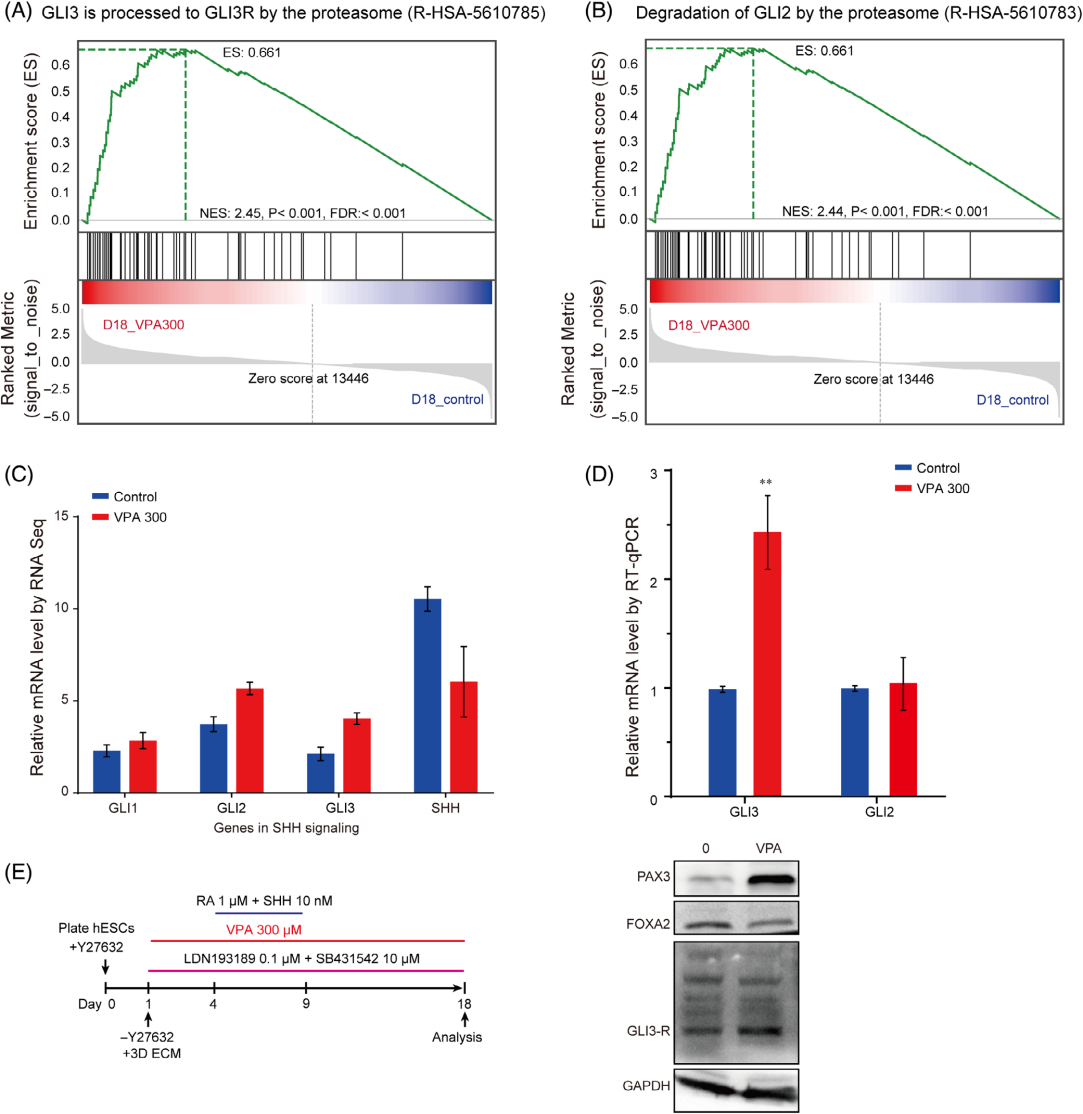
Degradation of GLI protein was induced by VPA in hNTOs. (A) GSEA of the pathway termed “GLI3 is processed to GLI3R by the proteasome” in VPA‐treated hNTOs compared with control, showing global upregulation of key genes involved in GLI3 processed to GLI3R. (B) GSEA of the pathway termed ‘Degradation of GLI2 by the proteasome’ in VPA‐treated hNTOs compared with control, showing global upregulation of key genes involved in degradation of GLI2. (C) The RNA‐seq data identified upregulation of Shh signalling pathway transcription factors *GLI1*, *GLI2*, *GLI3* mRNAs and downregulation of *SHH* mRNAs. (D) RT‐qPCR data showing the downregulation of *GLI3*, whereas *GLI2* expression was not affected. **p < 0.01. (E) Sample images of Western‐blot analysis showing the expression levels of Gli3‐R protein, ventral marker FOXA2 and dorsal marker PAX3 protein in organoids treated by 300 μM VPA for 18 days.

To test the global Shh pathway activity, we performed GSEA of the Reactome Pathway. Surprisingly, the results revealed upregulation of genes involved in pathways termed ‘GLI3 is processed to GLI3R by the proteasome’ (NES = 2.45, *p* < 0.001, FDR <0.001), and ‘Degradation of GLI2 by the proteasome’ (NES = 2.44, *p* < 0.001, FDR <0.001) in VPA‐treated hNTOs compared with controls (Figure 5A,B). These data show that VPA induced degradation of GLI2 and GLI3 proteins by the proteasome. There are 3 different GLI proteins mediate the transcriptional effects of Shh signalling, that is, GLI1, GLI2 and GLI3. GLI1 acts as a transcriptional activator. For GLI2 and GLI3, their full‐length proteins are transcriptional activators, whereas their truncated fragment (GLI2R and GLI3R) are transcriptional repressors of Shh signalling.^44^ It is reported that GLI2 functions mainly as a transcriptional activator, whereas GLI3 acts as a transcriptional repressor.^45^, ^46^ Many studies have shown that in the absence of Shh signalling, GLI3 is processed into a repressor GLI3R.^47^, ^48^ Therefore, results in this study illustrated that Shh signalling was inhibited by VPA‐induced GLI protein degradation. From RNA sequencing analysis, we identified upregulation of Shh signalling pathway transcription factors *GLI1*, *GLI2*, *GLI3* mRNAs and downregulation of *SHH* mRNAs (Figure 5C). Nonetheless, the RT‐qPCR results showed that *GLI3* mRNA was significantly upregulated, whereas *GLI2* mRNA was not affected in VPA‐treated hNTOs (Figure 5D). The Western‐blot analysis show that 300 μM VPA increased the Gli3‐R protein level (Figure 5E). To verify the degradation of GLI3 induced by VPA, SDS‐PAGE and liquid chromatography‐mass spectrometry/mass spectrometry (LC–MS/MS) were performed. The results show that GLI3 protein was identified in 300 μM VPA‐treated hNOT, whereas not in control group, indicating that the abundance of truncated GLI3 protein in VPA‐treated group was higher than that in control group (Figure S9). Consistent with RNA‐seq and Western blot results, the LC–MS/MS data confirmed that VPA induced GLI3 degradation, indicating the inhibition of Shh signalling pathway after VPA treatment.

The Western‐blot analysis also identified the upregulation of PAX3 (Figure 5E). PAX3 is a dorsal most marker of neural tube and induced by Wnt and BMP signalling.^24^ For the evaluation of BMP signalling, we conducted Western‐blot assays to determine the changes in P‐Smad 1/5 protein levels after VPA treatment. The results show that VPA slightly diminished P‐Smad 1/5 protein levels (Figure S10). We also used RNA‐seq data to investigate the expression levels of Smad1 and Smad5 genes. Consistent with the Western‐blot results, 300 μM VPA repressed the expression levels of Smad1 and Smad5 genes, but the inhibitory effect was not significant when compared with control. These results indicate that BMP signalling pathway was not activated by VPA. Therefore, the upregulation of PAX3 expression in VPA‐treated hNTOs confirmed that VPA indeed activated endogenous Wnt signalling even though VPA treatment duration was reduced. Taken together, we reasoned that VPA might enhance ventral patterning of hNTOs through both activating Wnt, which could antagonise Shh signalling by inducing GLI3 expression, and/or inhibiting Shh signalling by inducing GLI2 and GLI3 protein degradation.

### 2.6 Decreasing duration of VPA treatment enhances ventral patterning of hNTOs

**FIGURE 6:**
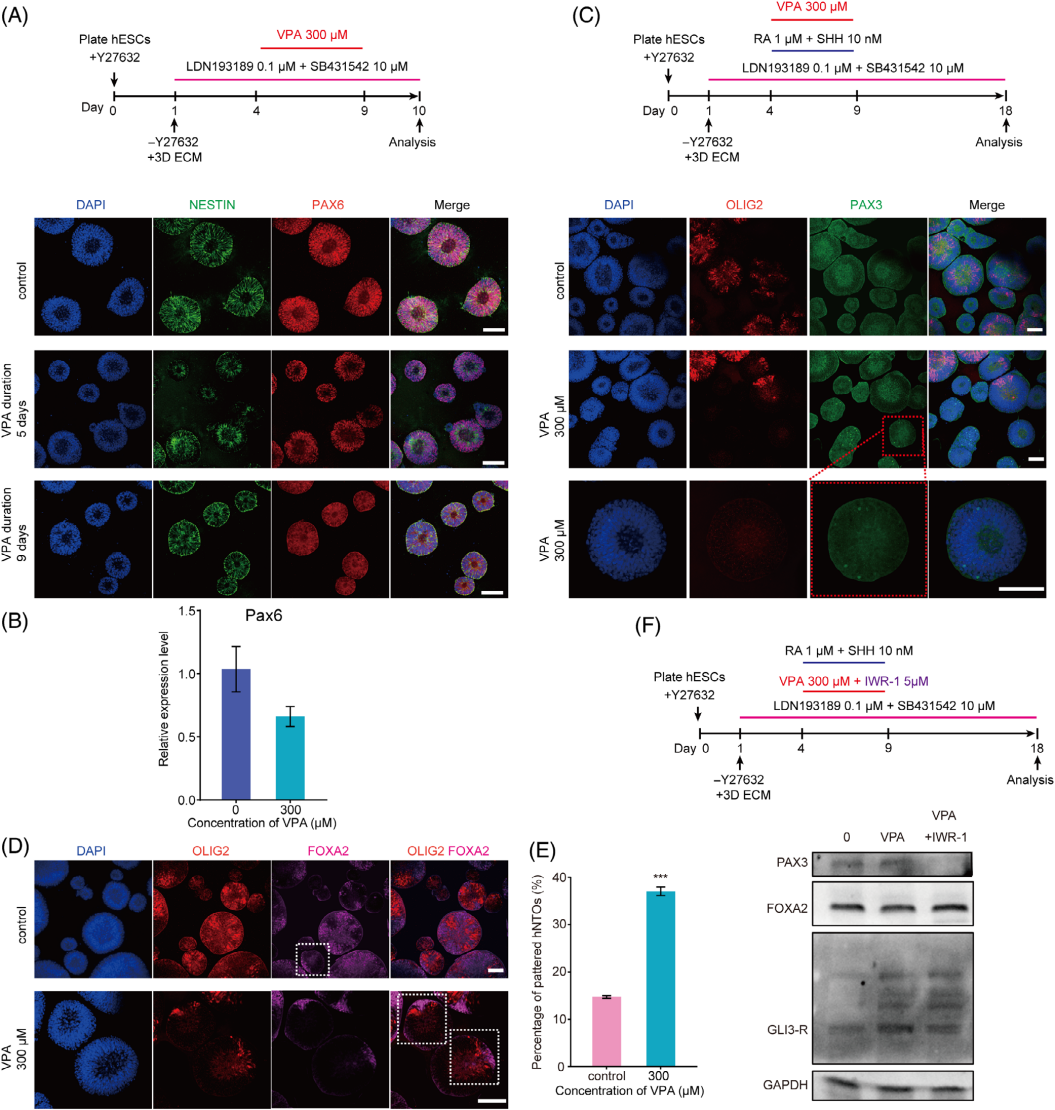
Decrease duration of valproic acid (VPA) enhances ventral patterning of hNTOs. (A) Representative image of primary hNTOs for PAX6 (red) and NESTIN (green) at day 10 when the duration time of VPA is reduced to 5 days. (B) RT‐qPCR data showing the downregulation of *PAX6*, but not significant. (C) Representative micrographs showing organoids obtained at day 18 stained for PAX3 and OLIG2 after the duration time of VPA was reduced to 5 days. The zoomed‐in images showing a magnified view of the area highlighted by the red square. (D) Representative confocal micrographs showing ventral patterned organoids obtained at day 18 stained for OLIG2 and FOXA2 after the duration time of VPA was reduced to 5 days. (E) Histogram graph showing percentages of ventral patterned organoids at days 18 after the duration time of VPA was reduced to 5 days. Data bars represent mean ± SEM. *p ≤ 0.05. (F) Sample images of Western‐blot analysis showing the expression levels of GLI3‐R protein, ventral marker FOXA2 and dorsal marker PAX3 protein in organoids treated by 300 μM VPA or 300 μM VPA + 5 μM IWR‐1 for 5 days. Scale bars, 50 μm (A, C and D). DAPI counterstained nuclei.

We further hypothesised that VPA could be applied to improve the efficiency of ventral patterning of hNTOs without affecting their formation. Hence, we next modulated VPA treatment duration time. To this end, VPA treatment was initiated on day 4 of hESCs differentiation, and the duration of VPA treatment was reduced to 5 days. On day 10, hNTOs were immunostained for PAX6, with data showing that most cells in hNTOs were positive for PAX6, similarly to untreated controls (Figure 6A). RT‐qPCR was conducted to confirm the effect of VPA short term exposure on PAX6 expression, and the data show that VPA reduced the expression of PAX6, but not significantly. These results indicate that VPA short term exposure had a weak influence on PAX6 expression (Figure 6B). At patterning stage, we observed PAX3 expression in control and VPA‐treated organoids fixed on day 18 (Figure 6C). Most importantly, ventral patterning efficiency of hNTOs remained significantly improved when initiation of VPA treatment was delayed and the duration of VPA reduced to 5 days, when compared with untreated controls (Figure 6D,E).

To confirm whether activation of the Wnt signalling pathway might contribute to short term exposure VPA‐induced gene expression changes, we treated hNTOs organoids with 0 μM VPA, 300 μM VPA, 300 μM VPA combined with Wnt signalling pathway inhibitor IWR‐1, and detected whether the expression of GLI3‐R protein, ventral marker FOXA2 and dorsal marker PAX3 protein was rescued by these molecules. The Western‐blot results show that IWR‐1 reversed the expression of the above proteins (Figure 6F). Immunostaining analysis also verified that IWR‐1 promoted the expression of OLIG2 and FOXA2 in the hNTOs organoid (Figure S11A). We further quantified the proportion of patterned hNOT in different conditions. With VPA (300 μM) and Wnt signalling pathway inhibitor IWR‐1 (5 μM) supplemented into neural differentiation medium from day 4 to day 9, 23.54% of hNOTs achieved proper ventral patterning at day 18, with spatially patterned OLIG2+ pMN domain and FOXA2+ FP domain at one pole of the hNOT (Figure S11B). This ratio is between the control and the Wnt inhibitor IWR‐1 group, indicating that the addition of the inhibitor IWR‐1 reversed part of the effect of VPA. We subsequently performed RT‐qPCR analysis to determine the levels of genes PAX3, OLIG2, FOXA2 and SHH. Consistent with Western‐blot results, the addition of the inhibitor IWR‐1 increased the expression levels of PAX3, OLIG2 and FOXA2, compared with control group (Figure S11C). The level's changes of the above genes are contrary to the results in the condition of VPA. Meanwhile, we detected the upregulation of SHH, indicating the upregulation of Shh signalling after inhibitor IWR‐1 treatment. Overall these results remarkably implicate the upregulation of Shh signalling after Wnt signalling pathway was inhibited, further proving that VPA could activate Wnt signalling pathway. All together, these findings suggest that activation of the Wnt signalling pathway maybe play an important role in VPA induced higher patterning efficiency, and that there is interaction between Shh signalling inhibition and Wnt signalling activation in VPA‐treated organoids.

## 3 DISCUSSION

Morphogen signalling, such as ventrally located Shh and dorsally located BMP and Wnt, controls the precise locations of specific progenitor regions of the human neural tube along the DV axis.^27^, ^49^ Wnt and BMP signalling favoured dorsal identities, and Shh induced ventral identity.^7^, ^50^ Therefore, chemicals that regulate Wnt, BMP, and Shh signalling activities could theoretically affect DV patterning of the neural tube. Recent studies have reported that small molecule VPA could activate Wnt signalling in neural stem cells.^9^, ^51^ Therefore, in this study we asked whether VPA could activate Wnt signalling in the hNTOs and thereby affect their regional DV patterning through Wnt signalling. In this report, we demonstrated VPA could promote ventral patterning of hNTOs by regulating Wnt signalling.

By modifying the protocol developed previously, we successfully induced PAX6+ hNTOs with a characteristic neural tube morphology and expression of NE markers (Figure 1). Most notably, our method enabled significant improved efficiency of ventral patterned hNTOs, which features the OLIG2+ pMN domain, NKX2.2+ p3 domain and FOXA2+ FP domain emerged in discrete, nonoverlapping regions (Figure 1). These results suggest that hNTOs recapitulated neural tube development and regional DV patterning to some extent. Compared with animal models, hNTO model is easy to operate and low‐cost, serving as an excellent experimental tool to study the impact of exogenous and genetic factors on neural tube development.^52^ For example, Abdel Fattah and colleagues report that active mechanical forces increased growth and lead to enhanced FP patterning of hNTOs.^15^ Moreover, spatial patterning of hNTOs was achieved by modulating RA and Shh signalling without exogeneous Wnt signals. Therefore, hNTO provides a suitable tool to study the role of VPA‐induced Wnt signalling in DV patterning of neural tube.

In this study, we found that treatment of hNTOs with 300 μM VPA resulted in almost complete disappearance of PAX6+ cells in early hNTOs (Figure 2). This experiment revealed that 300 μM VPA caused a pronounced inhibition of NE lineages in early hNTO development. This finding is consistent with previous reports that VPA disturbed early neuroepithelial development from human pluripotent stem cells.^53^, ^54^ Interestingly, as differentiation proceeds, VPA did not completely block NE differentiated into neural progenitor cells, but rather promoted ventral patterning efficiency of hNTOs. The quantification results showed that the efficiency of OLIG2+ FOXA2+ patterned hNTOs on day 18 increased from 15% to a maximum of 41% (Figure 3). Western‐blot and RT‐qPCR results revealed that expression of dorsal most marker PAX3 increased significantly in VPA‐treated hNTOs (Figure 3). PAX3 is strongly expressed in the dorsal half of the neural tube and regulated by Wnt signalling.^55^, ^56^ Given that many studies have shown that VPA could activate Wnt signalling, we reasoned that upregulated Wnt signalling might be involved in VPA induced dorsal fate and thereby promoted ventral patterning of hNTOs in this study.

Recent studies have demonstrated that VPA exposure in human brain organoids could activate Wnt signalling from RNA‐seq analysis.^12^, ^57^ Consistent with these findings, our transcriptome analysis of VPA‐treated hNTOs confirmed upregulation of Wnt signalling (Figure 4). To validate our RNA‐seq results, we characterised expression of dorsal genes *OLIG3*, *PAX3*, and *PAX7* induced by Wnt signalling and some other genes enriched in Wnt signalling term by RT‐qPCR analysis (Figure 4). As expected, the results were in line with the data of the bioinformatics analysis. Together, these findings suggested that Wnt signalling was indeed activated in VPA‐treated hNTOs. It is reported that Wnt signalling pathway restricts SHH patterning activity at the ventral regions of neural tube, and thus exhibiting opposite effects on ventral patterning with Shh signalling.^27^ Therefore, activating of the Wnt signalling pathway might be associated with changes in ventral patterning efficiency of hNTOs. Plenty evidence suggests that Wnt signalling affects ventral patterning by directly inducing expression of GLI3 to restrict SHH activity in ventral domains of the neural tube.^27^, ^58^ GLI3 is a potent antagonist of Shh signalling pathway.^47^, ^59^ Our transcriptome and RT‐qPCR analysis indeed identified upregulation of *GLI3* in VPA‐treated hNTOs (Figure 5). Our transcriptome analysis also revealed upregulation of genes involved in pathways termed “GLI3 is processed to GLI3R by the proteasome” and “Degradation of GLI2 by the proteasome” in VPA‐treated hNTOs. Therefore, our data support that VPA might have an impact on post‐transcriptional regulation, which might lead to misfolding and conformation changes of GLI proteins that are subsequently degraded by the proteasome system. Collectively, these results indicate that VPA enhances ventral patterning of hNTOs through activating intracellular Wnt signalling, which antagonises Shh signalling by inducing GLI3 expression, and/or inhibiting Shh signalling by promoting GLI protein degradation via a mechanism that remains to be fully elucidated in future studies.

Given our data showing that VPA exposure could enhance generation of patterned hNTOs by regulating Wnt and Shh signalling, we further speculated that VPA has the potential to be used to improve the patterning efficiency of hNTOs without affecting hNTO formation by adjusting its duration. After delaying addition time and reducing duration of VPA treatment, increased ventral patterning was observed in VPA‐treated hNTOs, similarly to hNTOs treated by VPA during the entire differentiation process (Figure 6). Importantly, we observed emergence of PAX3+ hNTOs, which were absent in untreated control hNTOs. Since PAX3 expresses in dorsal neural tube, the appearance of PAX3+ hNTOs further confirmed the activation of endogenous Wnt signalling in hNTOs, which directs dorsal differentiation in hNTOs. Recent years, small molecule VPA, has been developed for generating human expanded potential stem cells, motor neurons and even for inducing hair follicle regrowth by upregulating Wnt/β‐catenin.^8^, ^60^, ^61^ Therefore, VPA will be very valuable to fabricate patterned hNTOs and thus accelerate their application in tissue engineering, disease modelling and drug screening.

The main limitation of this study is that we did not compare the effect of Wnt signalling specific activators and VPA on the dorsal and ventral patterning of hNTOs. By optimising the concentration of Wnt signalling pathway activators or VPA, and SHH, higher patterning efficiency of patterned hNTOs can theoretically be achieved. These important studies will be the focus of our future work. In addition, we did not further confirm the efficiency of dorsal patterned hNTOs by adjusting the concentration and duration time of VPA and SHH. Moreover, we identified the upregulation of Wnt signalling, which located in the dorsal neural tube *in vivo,* by performing sequencing studies from hNTOs at patterning stage. As a results, it is possible that activated BMP signalling, which is known to regulate dorsal patterning of neural tube *in vivo*,^4^ may also play a role in improving the efficiency of DV pattered hNTOs. A recent study reports that Wnt and BMP signalling promotes FP patterning through inhibition effect.^62^ Therefore, it is necessary to explore the addition of BMP signalling activator in a certain time window on the influence of hNTOs patterning along DV axis. Furthermore, we did not proceed to elucidate the direct or indirect link between Shh signalling inhibition and Wnt signalling activation in VPA‐treated organoids. These important studies will help shed light on the interaction mechanism of BMP, Wnt and Shh signalling in neural tube DV patterning and further improve patterning efficiency of hNTOs.

## 4 CONCLUSIONS

In this work, we show that hNTOs have increased ventral patterning efficiency after 300 μM VPA treatment. Our transcriptome and RT‐qPCR data revealed that endogenous Wnt signalling was activated by VPA treatment. Furthermore, GSEA analysis of Reactome pathways identified upregulation of genes in promoting degradation of GLI3 to a truncated GLI3R and degradation of GLI1 and GLI2 by proteasome. These results indicated that Shh signalling in hNTOs was inhibited by VPA treatment. Even though it remains to be fully elucidated whether VPA could induce GLI protein degradation through direct interaction with GLI proteins or activation of related destruction signals, our findings strongly support that VPA could enhance ventral patterning efficiency of hNTOs by activating endogenous Wnt signalling in combination with its impact on inhibiting Shh signalling. Importantly, timing of VPA treatment could be finetuned to enhance ventral patterning of hNTOs without affecting their formation. Future studies should focus on tuning VPA and SHH treatment conditions to enhance DV patterning efficiency of hNTOs. In conclusion, this study demonstrated that VPA could be applied to modulate DV patterning of hNTOs by activating Wnt but inhibiting Shh signalling. The generation of more reproducible, DV patterned human neural tube organoids should facilitate their applications in tissue engineering, disease modelling and drug screening.

## 5 EXPERIMENTAL SECTION

### 5.1 Cell culture

hESC line H9 (Wicell) were maintained and cultured as previously described.^13^ Briefly, hESC were maintained on Matrigel hESC‐Qualified Matrix, LDEV‐free (Corning) coated dishes with mTeSR1 medium (STEMCELL Technologies). Cells were passaged every 5 days using Dissociation Buffer (Nuwacell Biotechnologies) and replated at a 1:6 to 1:8 split ratio onto a six‐well tissue culture plate (BD Biosciences). Cells used in this study had a passage number of <P50.

### 5.2 Fabrication of gel beds

The fabrication of Geltrex gel bed was performed as described previously.^13^, ^63^ Briefly, two 8‐ or 12‐mm diameter round glass coverslips were treated with air plasma (Harrick Plasma) for 2 min. One of the coverslips was soaked in poly‐(l‐lysine) solution (0.1 mg ml^−1^) (Sigma‐Aldrich) for 30 min and then in 1% glutaraldehyde solution (Electron Microscopy Sciences) for another 30 min. Meanwhile, another coverslip was coated with poly‐(l‐lysine)‐graft‐poly‐(ethylene glycol) (PLL‐g‐PEG, 0.1 mg mL; SuSoS) solution for 1 h. Undiluted Geltrex (Thermo Fisher Scientific) was then sandwiched between the two coverslips on ice and incubated at 37°C for 60 min. Gently separate the two closely fitting glass coverslips and the Geltrex gel bed was left on one of the coverslips. Finally, the gel bed was submerged in DMEM/F12 medium and incubated at 37°C overnight before plating cells at the following day.

### 5.3 Human NTO culture in gel bed

The differentiation culture for hNTOs was performed as described previously.^13^ In brief, hESCs were dissociated to single cells using Accutase (STEMCELL Technology) and resuspended in mTeSR1 containing the ROCK inhibitor Y27632 (10 μM; STEMCELL Technology). Singly dissociated hESCs were plated onto Gelbed at an initial cell seeding density of 30 × 10^3^ cells/cm^2^ and cultured overnight. After 24 h (day 1), culture medium was switched to fresh N2SM1 induction medium [Advance DMEM/F12 (Gibco):Neurobasal medium (1:1; Gibco), 0.5× N2 Supplement‐A (STEMCELL Technology), 0.5× SM1 Neuronal Supplement (STEMCELL Technology), 1× nonessential amino acids (Gibco), 2 mM l‐glutamine (Gibco), and 0.1 mM β‐mercaptoethanol (Sigma‐Aldrich)] supplemented with the TGF‐β pathway inhibitor SB (10 μM; STEMCELL Technologies) and the BMP inhibitor LDN (0.1 μM; STEMCELL Technologies) to initiate neural induction. For 3D organoid generation, this neural induction medium was supplemented with 2% (v/v) Geltrex. The neural induction medium with 2% (v/v) Geltrex supplement was fully refreshed every 2 days until endpoint at day 10.

For patterning of neural tube organoid, all‐trans RA (1 μM; STEMCELL Technologies) and recombinant human SHH (10 nM; PeproTech) were supplemented into neural induction medium from day 4 to day 9. For investigating effect of VPA on neural tube organoids generation, different concentrations of VPA (Sigma, P4543, purity >98.0%) dissolved in DMEM/F12 (Gibco) was supplemented into N2SM1 neural induction medium. For the Wnt signalling antagonist treatment, IWR‐1 (5 μM, selleck) was also added to the medium from day 4 to day 9, and organoids were collected at day 18.

### 5.4 Cell viability assay in 3D organoids

For examination of the cell viability upon VPA treatment, hESCs suspended in 100 μL mTeSR1 complete medium were seeded onto 96‐well plates coated by thick Geltrex. At the following day, media were replaced with fresh neural induction medium supplemented with 2% (v/v) Geltrex and different concentrations of VPA. 48 h later, cells were incubated with fresh neural induction medium containing CCK‐8 (Solarbio). After incubation at 37°C with 5% CO_2_ for 3 h, the absorbance values of the cells at 450 nm were measured using Synergy H1 microplate reader (Bio Tek).

### 5.5 Immunocytochemistry

4% paraformaldehyde (Shanghai Yuanye Bio‐Technology) was used to fix hNTOs for 1 h and then permeabilised with 0.1% SDS (dissolved in D‐PBS) solution at room temperature for 3 h. Blocking was performed in 2% donkey serum solution (Solarbio) at 4°C overnight. Primary antibodies (Table S1) were suspended in blocking solution and applied to hNTOs for 24 h. hNTOs were then incubated with donkey‐raised secondary antibodies at 4°C for another 24 h. DAPI (Invitrogen) was used to visualise nuclei.

### 5.6 Image acquisition and analysis

For quantification analysis, fluorescence images were recorded by the Operetta CLS high content analysis system (PerkinElemer, Britain) equipped with high‐power 8× LED illumination, and 20×, 63× water‐immersion objectives. Confocal representative fluorescence images were obtained using a confocal microscope (Leica SP8 DIVE, Leica Microsystems) operated on confocal mode with 20×, 63× oil‐immersion objectives. For visualising multiple hNTOs in one fluorescence image, background of some images was removed in Photoshop.

### 5.7 Organoid size evaluation

To evaluate hNTO size, images stained with DAPI were analysed using Operetta CLS High Content Imaging System (PerkinElmer, Britain) by automatic tracing of organoid borders to obtain an area which is then converted to an equivalent diameter.

### 5.8 RNA Extraction and qRT‐PCR Analysis

Each total RNA sample was extracted using TRIzol Reagent (Invitogen), after which 1 μg of total RNA was used for reverse transcription by Hifair III 1st Strand cDNA Synthesis SuperMix (YEASEN, Shanghai, China) according to the manufacturer's protocol. Amplification was performed with the Hieff qPCR SYBR Green Master Mix (No Rox) (YEASEN, Shanghai, China). qPCR was performed on the CFX Connect Real‐Time System (Bio‐Rad). The data were normalised to GAPDH expression and expression levels are shown as mean values ± SEM. Primer sequences are listed in Table S2.

### 5.9 Western blot

Cells were lysed with RIPA Lysis Buffer (Biosharp) containing Pierce Protease Inhibitor Tablets (Thermo Scientific) and PMSF (Beyotime). Equal of total proteins were loaded in SDS‐polyacrylamide electrophoresis gel and then transferred to Immun‐Blot PVDF Membrane (BIO‐RAD). PVDF membranes were blocked with 5% non‐fat milk in TBS‐T for 1 h and then with primary antibodies (Table S1) overnight at 4°C. After incubation with appropriate horseradish peroxidase (HRP)‐labelled secondary antibody for 1 h at room temperature, chemiluminescent signals were detected by Tanon‐5200 (Tanon, China) using ECL Western Blotting Substrate (Tanon, China). To verify the degradation of GLI3, the bands of GLI3R protein in SDS‐PAGE were analysed by liquid chromatography‐mass spectrometry/mass spectrometry (OE Biotech, Shanghai, China). The cytoplasmatic and nuclear proteins were extracted using the Nuclear and Cytoplasmic Protein Extraction Kit (BL670A, Biosharp Life Science, China).

### 5.10 RNA‐seq and analysis

Total RNA was extracted using the TRIzol reagent (Invitrogen, CA, USA). VAHTS Universal V6 RNA‐seq Library Prep Kit was used for preparation of the RNA‐seq library. RNA‐seq was performed using the llumina Novaseq 6000 platform and 150 bp paired‐end reads were generated. Raw reads of fastq format were firstly processed with fastp1 for Illumina adapter trimming and removal of low‐quality reads. For gene expression analysis, sequence FASTQ files were mapped to the reference human genome using HISAT2. FPKM (fragments per kilobase of the exon model per million mapped fragments) of each gene was calculated and the read counts of each gene were obtained by HTSeq‐count. The transcriptome sequencing and analysis were conducted by OE Biotech Co., Ltd. (Shanghai, China).

### 5.11 Statistical analysis

GraphPad Prism was used for all statistical analyses. All data are shown as mean values ± SEM. The comparison of data among treatment groups was analysed by one‐way ANOVA. Statistical analysis on the RT‐qPCR data was performed using two‐sided unpaired Student's *t* tests. A *p* value of <0.05 was considered statistically significant for all tests.

## AUTHOR CONTRIBUTIONS

Conceptualization, Y.Z. and F.Z; methodology, Y.Z., F.Z. and J.F.; investigation, F.Z., H.N., X.L., and J. X.; formal analysis, Y.Z. and F.Z; visualisation, F.Z., H.N., X.L., and J. X.; writing—original draft, Y.Z., F.Z and J.F.; Writing—review and editing, Y.Z., J.F. and L.W.; resources, Y.Z. and L.W.; supervision, Y.Z. and L.W.; funding acquisition, Y.Z. and L.W.

## FUNDING INFORMATION

National Natural Science Foundation of China [grant number 32101106]; Joint Funds of the National Natural Science Foundation of China [grant number U22A20406]; Natural Science Foundation of Anhui Province [grant number 109065895022]; Advanced Innovative Programs for the Returned Overseas Chinese Scholars in Anhui province [grant number 2020LCX002].

## CONFLICT OF INTEREST STATEMENT

The authors declare that they have no competing interests.

## Supporting information


**Table S1.** List of primary antibodies used in immunofluorescence (IF) and Western‐blot.
**Table S2.** List of primers used in RT‐qPCR.

**Figure S1.** The PAX3 + FOXA2+ patterning in a hNTO.

**Figure S2.** The cell viability assay of 3D organoids treated by different concentration of VPA.

**Figure S3.** Principle component analysis (PCA) and sample clustering illustrating variance within and across VPA treated and control organoids.

**Figure S4.** Volcano plot of DEGs analysis between VPA treated and control hNTOs.

**Figure S5.** KEGG enrichment results of down‐regulated DEGs.

**Figure S6.** The graph shows RNA‐seq results (normalised FPKM) of gene sets enriched in WNT signalling.

**Figure S7.** The analysis of β‐catenin expression after treatment of hNOTs with 300 μM VPA.

**Figure S8.** The graph shows RNA‐seq results (normalised FPKM) of HOX (A–D) genes along AP axis.

**Figure S9.** The secondary mass spectrometry of GLI3R protein band in VPA‐treated organoids.

**Figure S10.** The BMP signalling pathway was not activated by VPA.

**Figure S11.** The effect of Wnt inhibition IWR‐1 on the ventral patterning of hNTOs.

## Data Availability

The accession number for the transcriptomic data reported in this article is available at NCBI Trace and Short‐Read Archive (SRA): PRJNA1068746. All other data supporting the findings of this study are available from the corresponding author upon reasonable request. The original contribution data required to reanalyse the data reported in this article is available from the lead contact upon request.
