# Supplementary material for: Small molecule valproic acid enhances ventral patterning of human neural tube organoids by regulating Wnt and Shh signalling
Source: Cell Prolif. 2024 Aug 20;58(1):e13737. doi: 10.1111/cpr.13737 (PMC11693559; doi:10.1111/cpr.13737)
Supplement: Supplementary file 1 — Table S1. List of primary antibodies used in immunofluorescence (IF) and Western‐blot. Table S2. List of primers used in RT‐qPCR. [file CPR-58-e13737-s012.docx]

Supporting Information

**Small molecule valproic acid enhances generation of patterned human neural tube organoids by regulating** **Wnt and Shh signaling**

**Yuanyuan zheng ^1, 5^, Fangrong Zhang ^1, 5^,** **Haifeng Nie^1^, Xinyu Li^1^, Jiali Xun^1^, Jianping Fu^2, 3, 4, *^ and Lijun Wu ^1, 6, *^**

^1^Information Materials and Intelligent Sensing Laboratory of Anhui Province, Institute of Physical Science and Information Technology, Anhui University, Hefei, Anhui 230601, China;

^2^Department of Mechanical Engineering, University of Michigan, Ann Arbor, MI 48109, USA;

^3^Department of Cell & Developmental Biology, University of Michigan Medical School, Ann Arbor, MI 48109, USA;

^4^Department of Biomedical Engineering, University of Michigan, Ann Arbor, MI 48109, USA.

^5^These authors contributed equally to this work.

^6^Lead Contact

^⁎^ Corresponding authors: [ljw@ipp.ac.cn](mailto:ljw@ipp.ac.cn) (L.W.) and [jpfu@umich.edu](mailto:jpfu@umich.edu) (J.F.)

**Figure captions**

Figure S1. The PAX3+FOXA2+ patterning in a hNTO.

Figure S2: The cell viability assay of 3D organoids treated by different concentration of VPA.

Figure S3: Principle component analysis (PCA) and sample clustering illustrating variance within and across VPA treated and control organoids.

Figure S4: Volcano plot of DEGs analysis between VPA treated and control hNTOs.

Figure S5: KEGG enrichment results of down-regulated DEGs.

Figure S6: The graph shows RNA-seq results (normalized FPKM) of gene sets enriched in WNT signaling.

Figure S7. The analysis of β-catenin expression after treatment of hNOTs with 300 μM VPA.

Figure S8. The graph shows RNA-seq results (normalized FPKM) of HOX (A–D) genes along AP axis.

Figure S9. The secondary mass spectrometry of GLI3R protein band in VPA treated organoids.

Figure S10. The BMP signaling pathway was not activated by VPA.

Figure S11. The effect of Wnt inhibition IWR-1 on the ventral patterning of hNTOs

**Table captions**

Supplementary Table 1. List of primary antibodies used in immunofluorescence (IF) and Western-blot.

Supplementary Table 2. List of primers used in RT-qPCR.

**Figure S1**

**
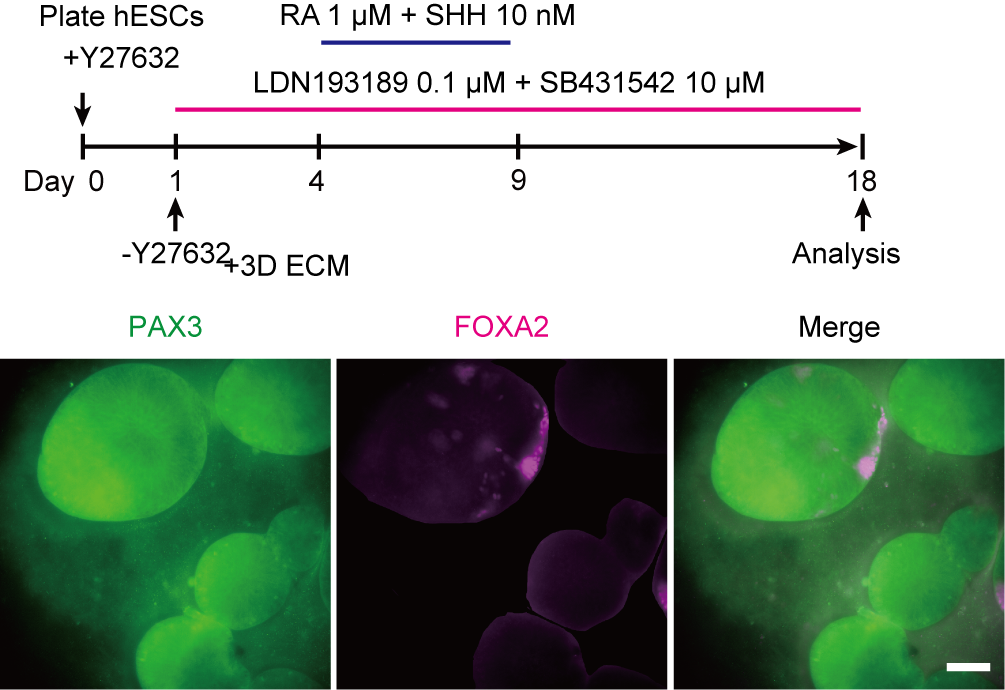
**

**Figure S1. The PAX3+FOXA2+ patterning in a hNTO.**

**Figure S2**


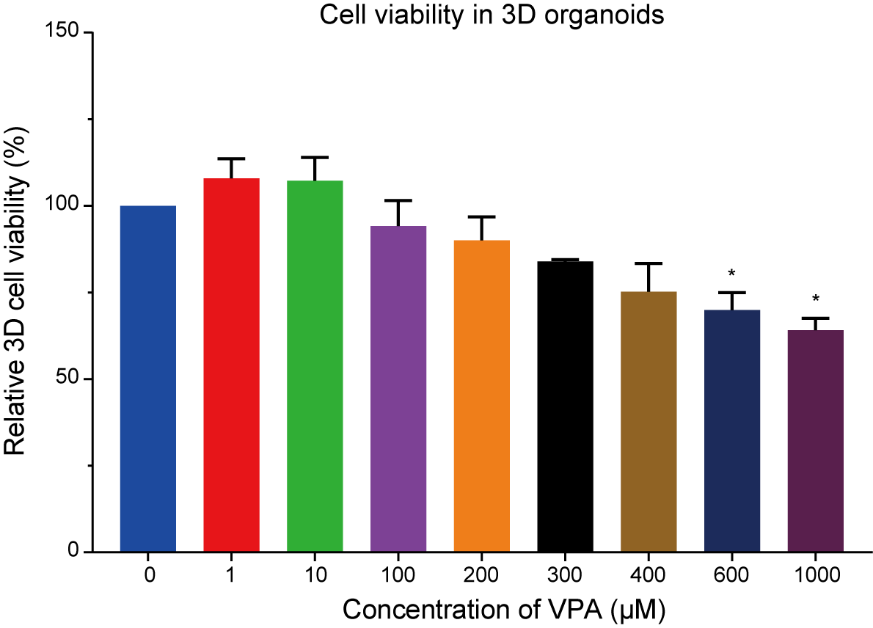


**Figure S2. The cell viability assay of 3D organoids treated by different concentration of VPA.**

**Figure S3**


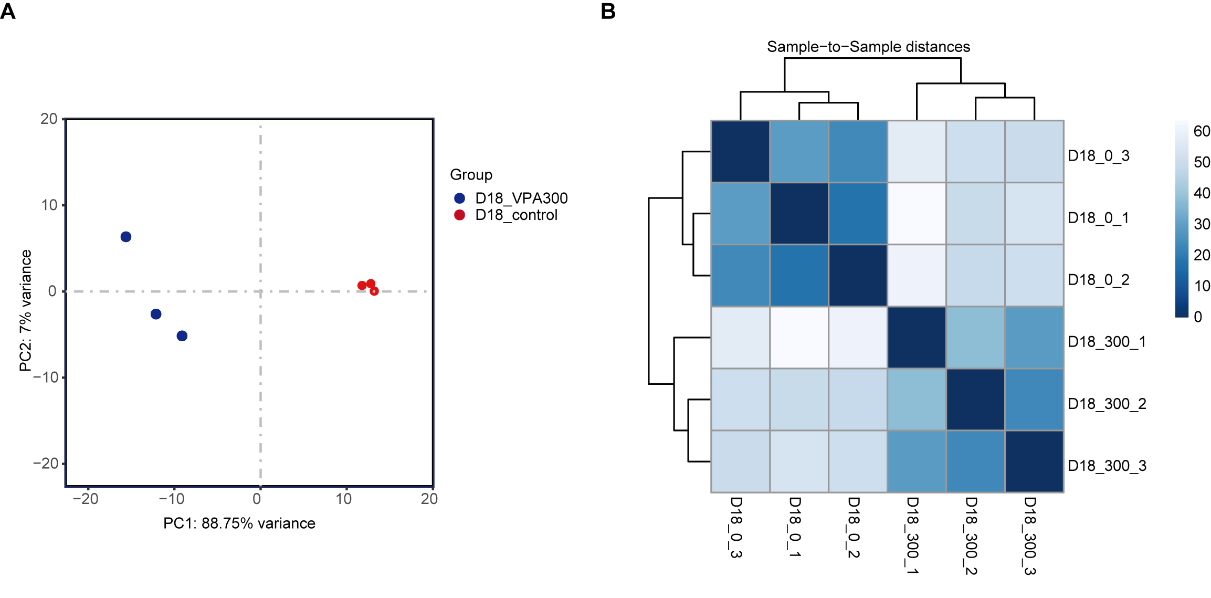


**Figure S3. Principle component analysis (PCA) and** **sample clustering illustrating variance within and across VPA treated and control organoids.**

(A) High variance (88.75 %) is observed between control vs 300 μM VPA treated organoids, showing a distinct segregation of transcripts for VPA treated and control hNTOs.

(B) Hierarchical sample clustering shows that replicates in control or 300 μM VPA treated organoids cluster strongly together, indicating low variance between biological replicates.

**Figure S4**


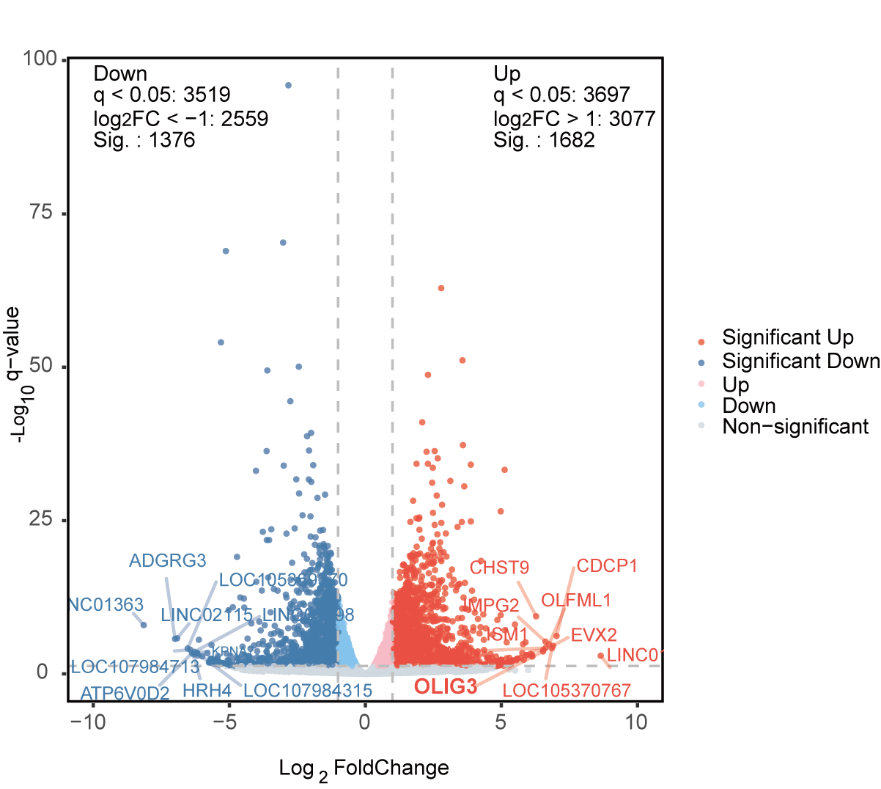


**Figure S4. Volcano plot of DEGs analysis between VPA treated and control hNTOs.** Dashed line indicates |log2 Fold Change| = 1. Up-regulated genes are shown in red, whereas down-regulated genes are shown in blue. p values < 0.05.

**Figure S5**


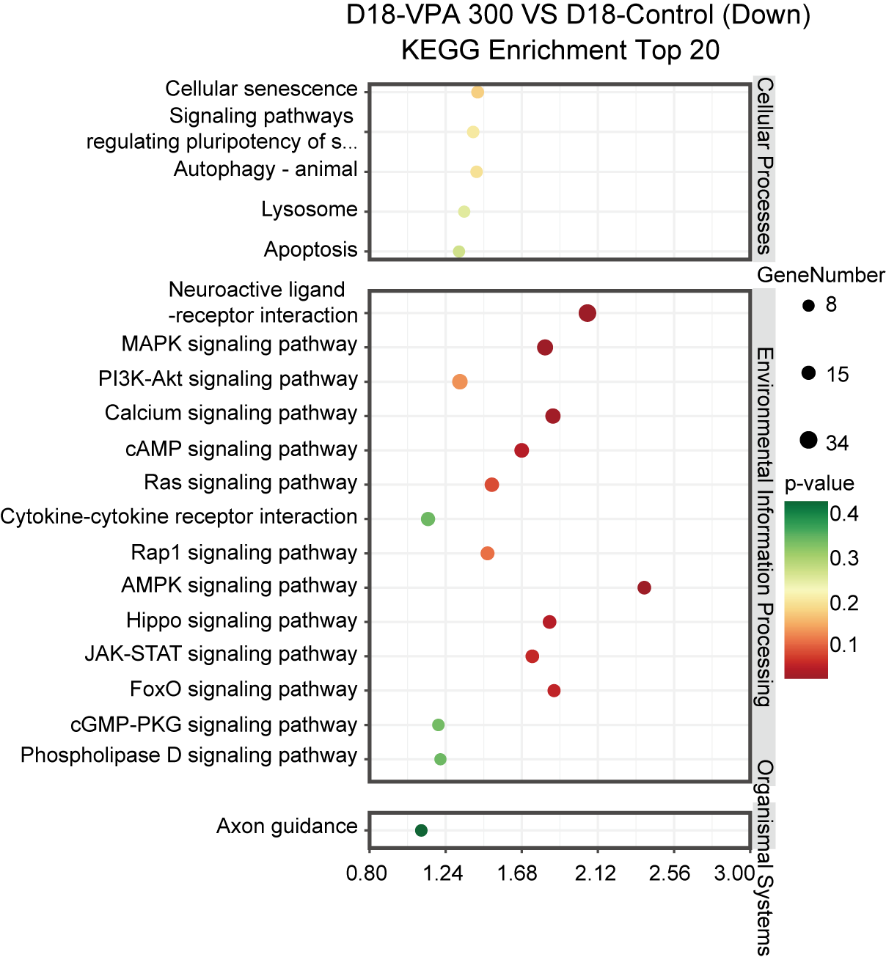


**Figure S5. KEGG enrichment results of down-regulated DEGs. The bubble plot showes the top 20 significant pathways in the KEGG analysis.** The analysis revealed downregulated DEGs were mainly associated with PI3K-Akt signaling, Hippo signaling, neuroactive ligand-receptor interaction and ECM-receptor interaction pathways in VPA treated hNTOs.

**Figure S6**


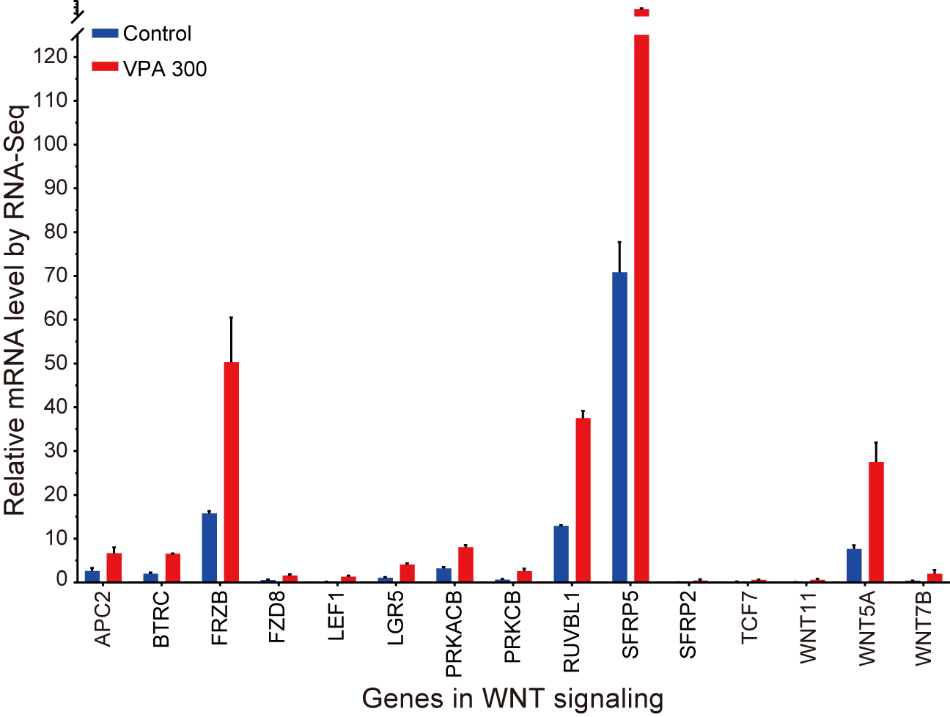


**Figure S6. The graph shows RNA-seq results (normalized FPKM) of gene sets** **enriched in Wnt signaling.**

**Figure S7**


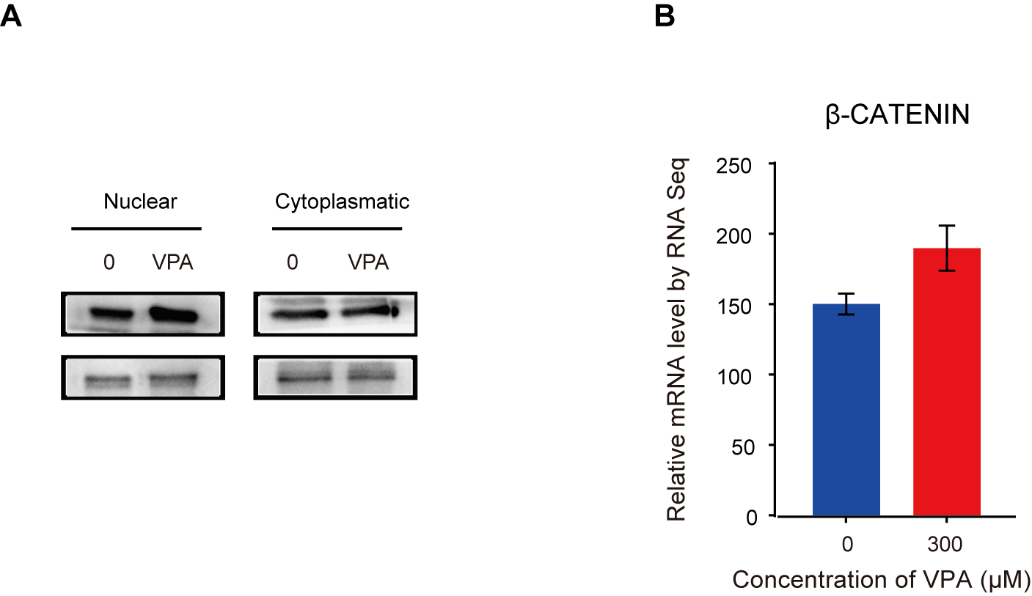


**Figure S7. The analysis of β-catenin expression after treatment of hNOTs with 300 μM VPA.** (A) Western blot analysis of β-catenin expression in nuclear and cytoplasmatic fractions after treatment of hNOTs with 300 μM VPA during the entire differentiation process. (B) The mRNA level of β-CATENIN using RNA-seq data.

**Figure S8**

**
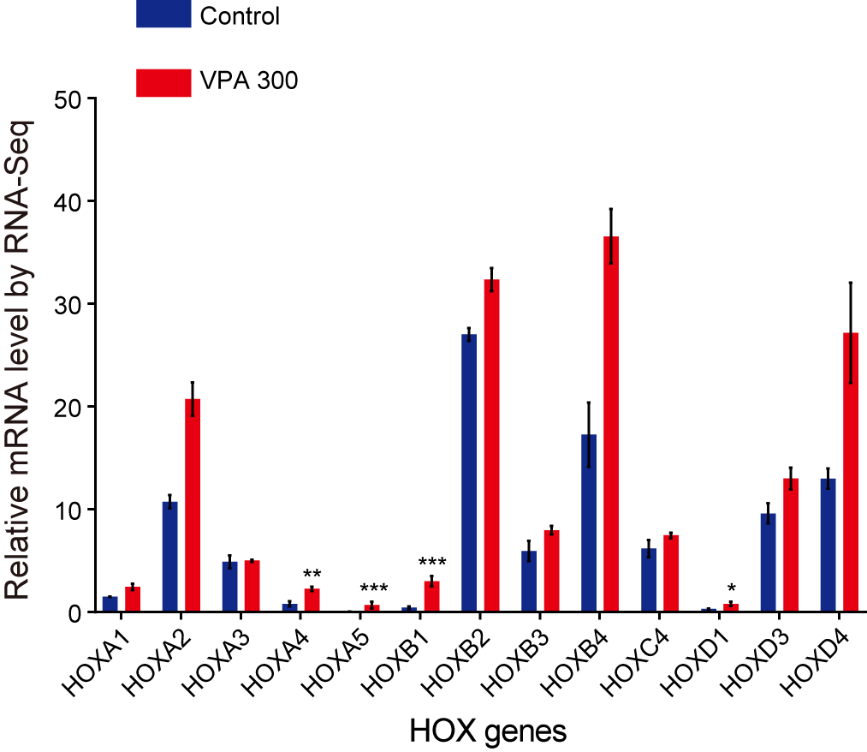
**

**Figure S8. The graph shows RNA-seq results (normalized FPKM) of HOX (A–D) genes along AP axis.**

**Figure S9**

**
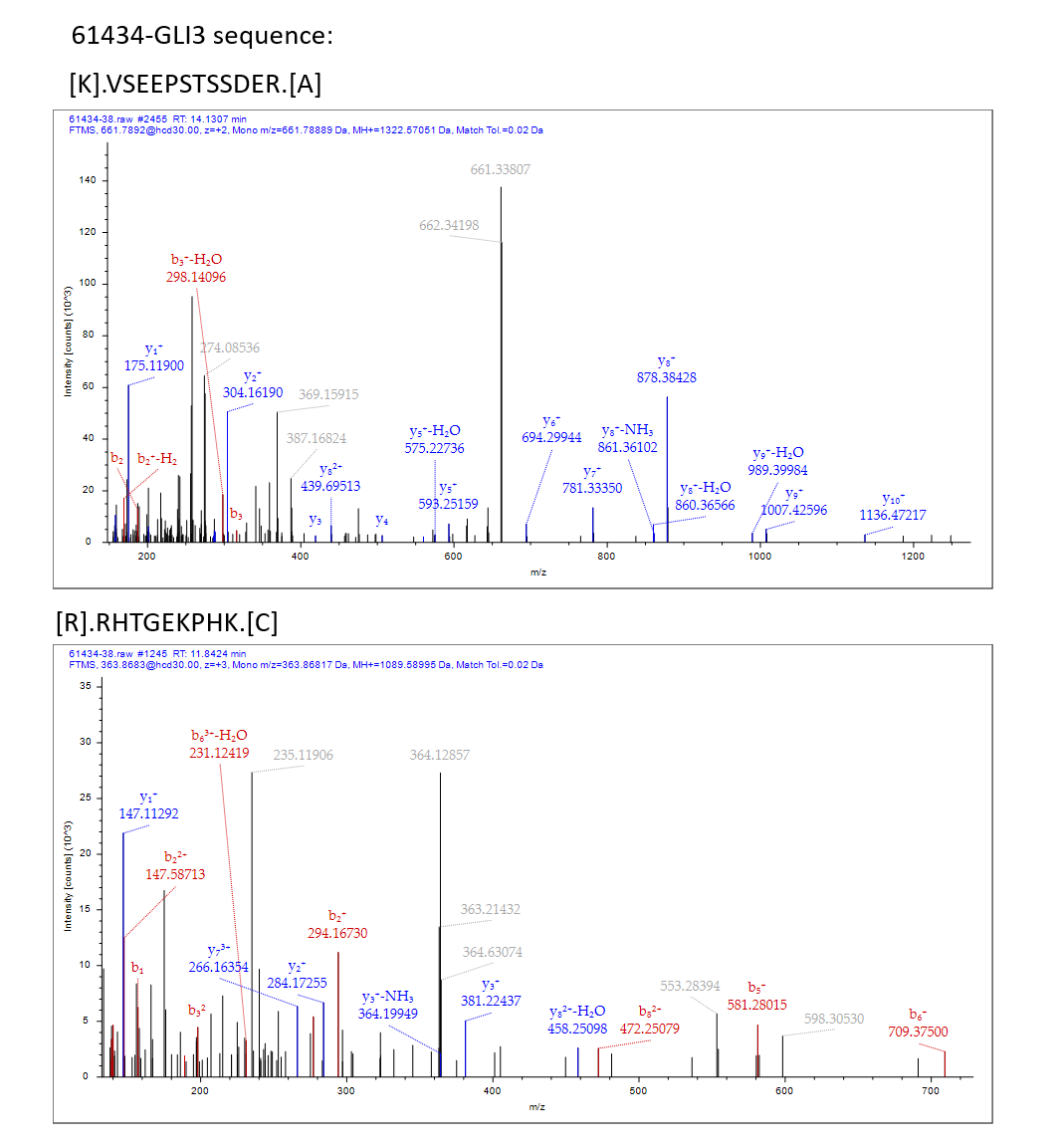
**

**Figure S9. The secondary mass spectrometry of GLI3R protein band in VPA treated** **organoids.**

**Figure S10**

**
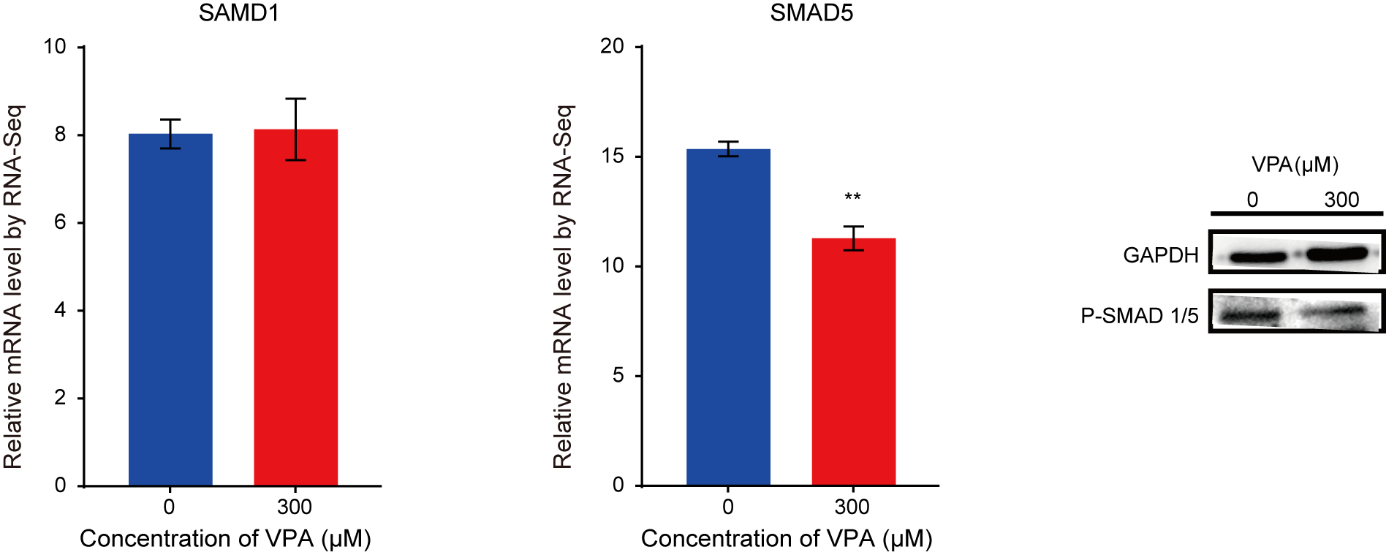
**

**Figure S10.** **The BMP signaling pathway was not activated by VPA.**

**Figure S11**


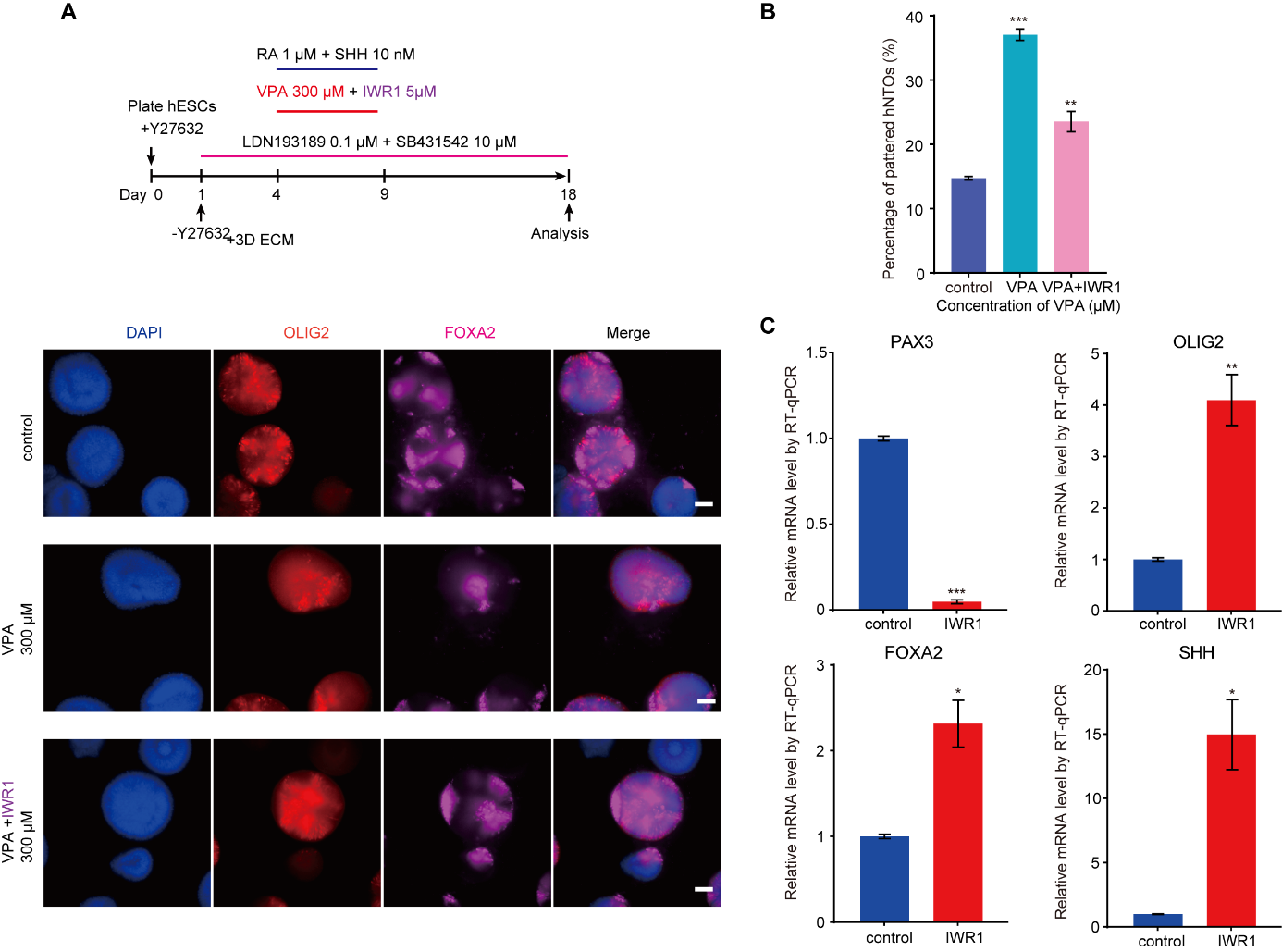


**Figure S11. The effect of Wnt inhibition IWR-1 on the ventral patterning of hNTOs.** (A) Representative micrographs showing VPA/IWR-1 treated hNOT at day 18 stained for ventral markers as indicated. (B) Percentages of patterned cysts from control, 300 μM VPA, and 300 μM VPA + 5 μM IWR-1 for 5 days. (C) RT-qPCR data showing the upregulation of PAX3, OLIG2, FOXA2, and SHH in organoids treated by 300 μM VPA + 5 μM IWR-1 for 5 days. * p ≤ 0.05, ** p < 0.01, *** p < 0.001. Data in B and C represent the mean ± SEM.

**SUPPLEMENTARY TABLES**

**Supplementary Table 1.** **List of primary antibodies used in immunofluorescence (IF) and Western-blot (WB).**

| Protein | Vendor | Catalog number | Dilution |
| --- | --- | --- | --- |
| NANOG | Abcam | ab109250 | 1:300 (IF) |
| OCT4 | Santa Cruz Biotech | sc-5279 | 1:500 (IF) |
| PAX6 | Biolegend | 901302 | 1:500 (IF); 1:500 (WB) |
| NESTIN | Santa Cruz Biotech | sc-23927 | 1:500 (IF) |
| OLIG2 | GeneTex | GTX132732 | 1:500 (IF); 1:500 (WB) |
| FOXA2 | R&D Systems | AF2400 | 1:500 (IF); 1:500 (WB) |
| PAX3 | DSHB | Supernatant | 1:50 (IF); 1:500 (WB) |
| NKX2.2 | R&D Systems | AF2400 | 1:500 (IF); 1:500 (WB) |
| Gli3 | R&D Systems | AF3690-SP | 0.2 µg/mL (WB) |
| Phospho-SMAD1/5 | Cell Signaling Technology | #9516 | 1:1000 (WB) |
| β-catenin | Cell Signaling Technology | 8480T | 1:1000 (WB) |
| GAPDH | ZENBIO | 380626 | 1:1000 (WB) |

**Supplementary Table 2. List of primers used in RT-qPCR.**

| Gene | Primer Sequences (5' -> 3') | Reference |
| --- | --- | --- |
| GAPDH | Forward:ACATCAAGAAGGTGGTGAAGCAGG | PrimerBank |
|  | Reverse: AGCTTGACAAAGTGGTCGTTGAGG | PrimerBank |
| PAX6 | Forward: TCTTTGCTTGGGAAATCCG | PrimerBank |
|  | Reverse: CTGCCCGTTCAACATCCTTAG | PrimerBank |
| PAX3 | Forward: GTGCCGTCAGTGAGTTCCATC | PrimerBank |
|  | Reverse: CTGTTCTGCTGTGAAGGTGGTT | PrimerBank |
| OLIG2 | Forward: GGGCCACAAGTTAGTTGGAA | Maury et al.^1^ |
|  | Reverse: GAGGAACGGCCACAGTTCTA | Maury et al.^1^ |
| NKX2.2 | Forward: AAACCATGTCACGCGCTCA | PrimerBank |
|  | Reverse: GGCGTTGTACTGCATGTGCT | PrimerBank |
| FOXA2 | Forward: GCAGAGACGCAAGGGAGAAGAA | PrimerBank |
|  | Reverse: GCAACAACAGCAATGGAGGAGAA | PrimerBank |
| OLIG3 | Forward: GAGCAGGACCTACAGCAGTT | PrimerBank |
|  | Reverse: GCCAACCAGCCTCTTCATCT | PrimerBank |
| PAX7 | Forward: TCGGATGTGGAGTCGGAACCT | PrimerBank |
|  | Reverse: TCCTGCCTGCTTACGCCAAC | PrimerBank |
| Gli2 | Forward: GCTGGCTGACCTCAAGGAAGAT | PrimerBank |
|  | Reverse: CCGTGGATGTGCTCGTTGTTG | PrimerBank |
| Gli3 | Forward: CCATCCGCTGTGCTCTAATCTG | PrimerBank |
|  | Reverse: AGGCTGTCCACTGTGCTTGTC | PrimerBank |
| LEF1 | Forward: AATGAGAGCGAATGTCGTTGC | PrimerBank |
|  | Reverse: GCTGTCTTTCTTTCCGTGCTA | PrimerBank |
| RUVBL1 | Forward: TGAGGAGGCACTGAACCACCTG | PrimerBank |
|  | Reverse: TTGGCGGAGGACTTGGCATCA | PrimerBank |
| WNT5A | Forward: AGGTCAACAGCCGCTTCAACTC | PrimerBank |
|  | Reverse: CGTAGCAGCACCAGTGGAACTT | PrimerBank |
| WNT7B | Forward: TCCTTCGTTCCCTCCCTCATCC | PrimerBank |
|  | Reverse: GGTAGGTCCTTGTGCCACTCTG | PrimerBank |

**Reference**

1. Maury, Y.; Côme, J.; Piskorowski, R. A.; Salah-Mohellibi, N.; Chevaleyre, V.; Peschanski, M.; Martinat, C.; Nedelec, S., (2015). Combinatorial analysis of developmental cues efficiently converts human pluripotent stem cells into multiple neuronal subtypes. Nature biotechnology *33*, 89-96. <https://doi.org/https://doi.org/10.1038/nbt.3049>.
