# Supplementary figures and images for: Small molecule valproic acid enhances ventral patterning of human neural tube organoids by regulating Wnt and Shh signalling
Source: Cell Prolif. 2024 Aug 20;58(1):e13737. doi: 10.1111/cpr.13737 (PMC11693559; doi:10.1111/cpr.13737)

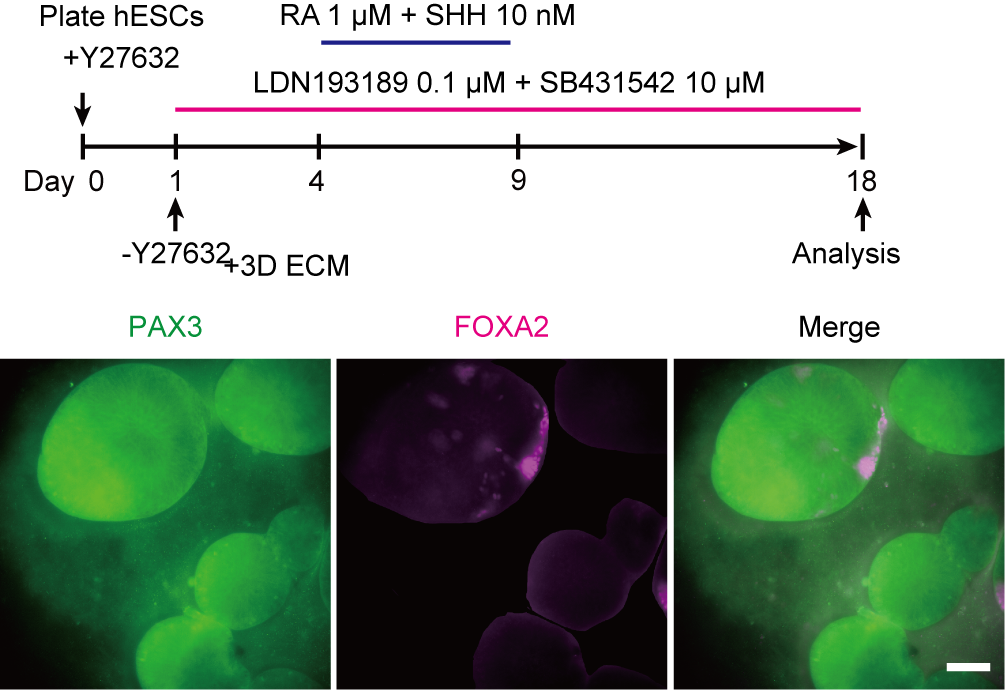

Supplement: Supplementary file 2 — Figure S1. The PAX3 + FOXA2+ patterning in a hNTO. [file CPR-58-e13737-s006.tif]

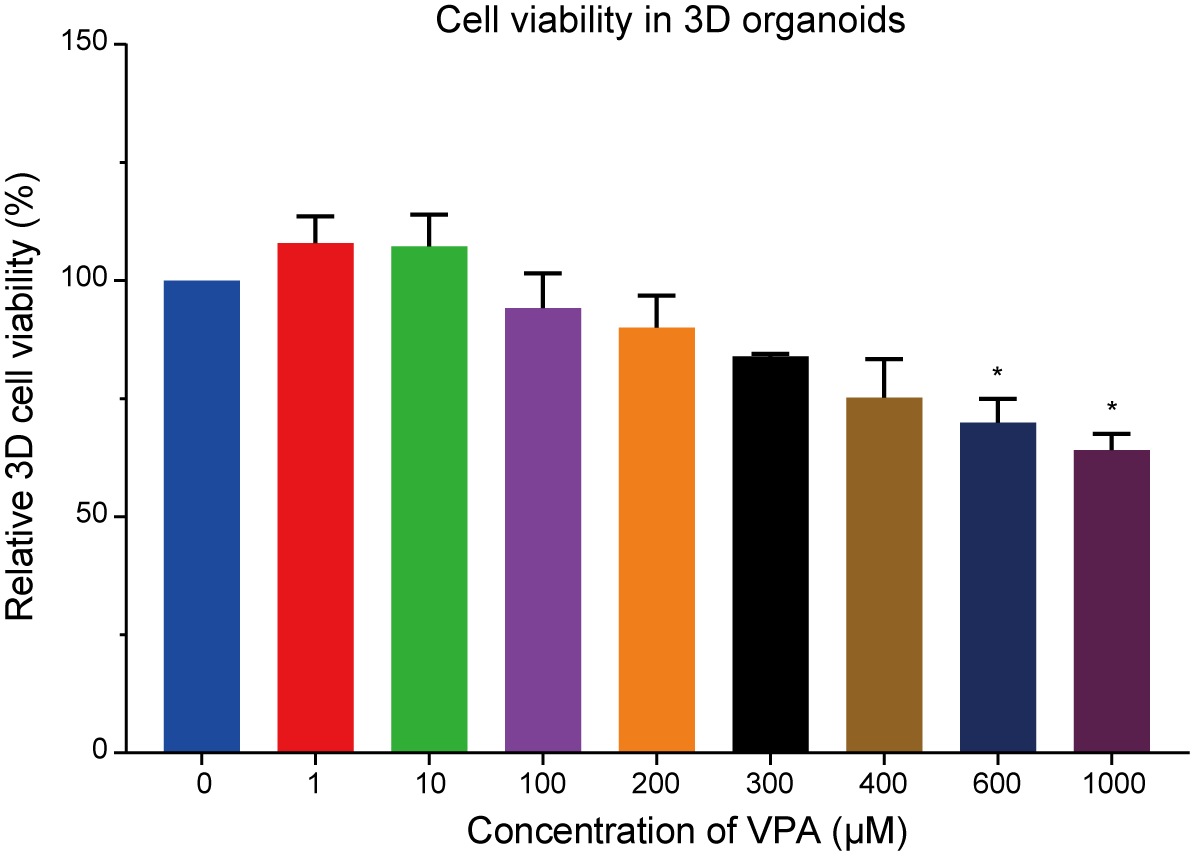

Supplement: Supplementary file 3 — Figure S2. The cell viability assay of 3D organoids treated by different concentration of VPA. [file CPR-58-e13737-s005.tif]

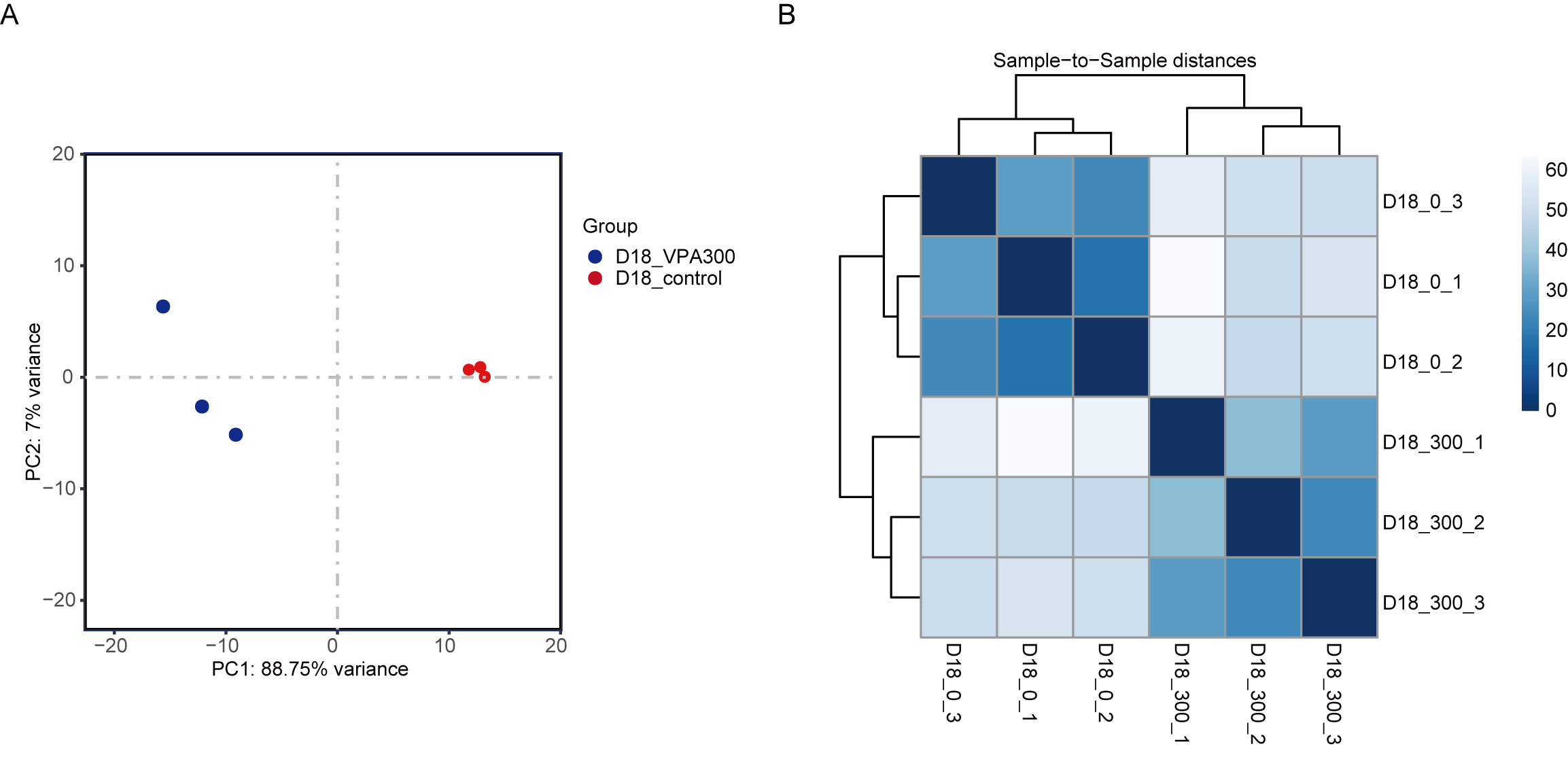

Supplement: Supplementary file 4 — Figure S3. Principle component analysis (PCA) and sample clustering illustrating variance within and across VPA treated and control organoids. [file CPR-58-e13737-s009.tif]

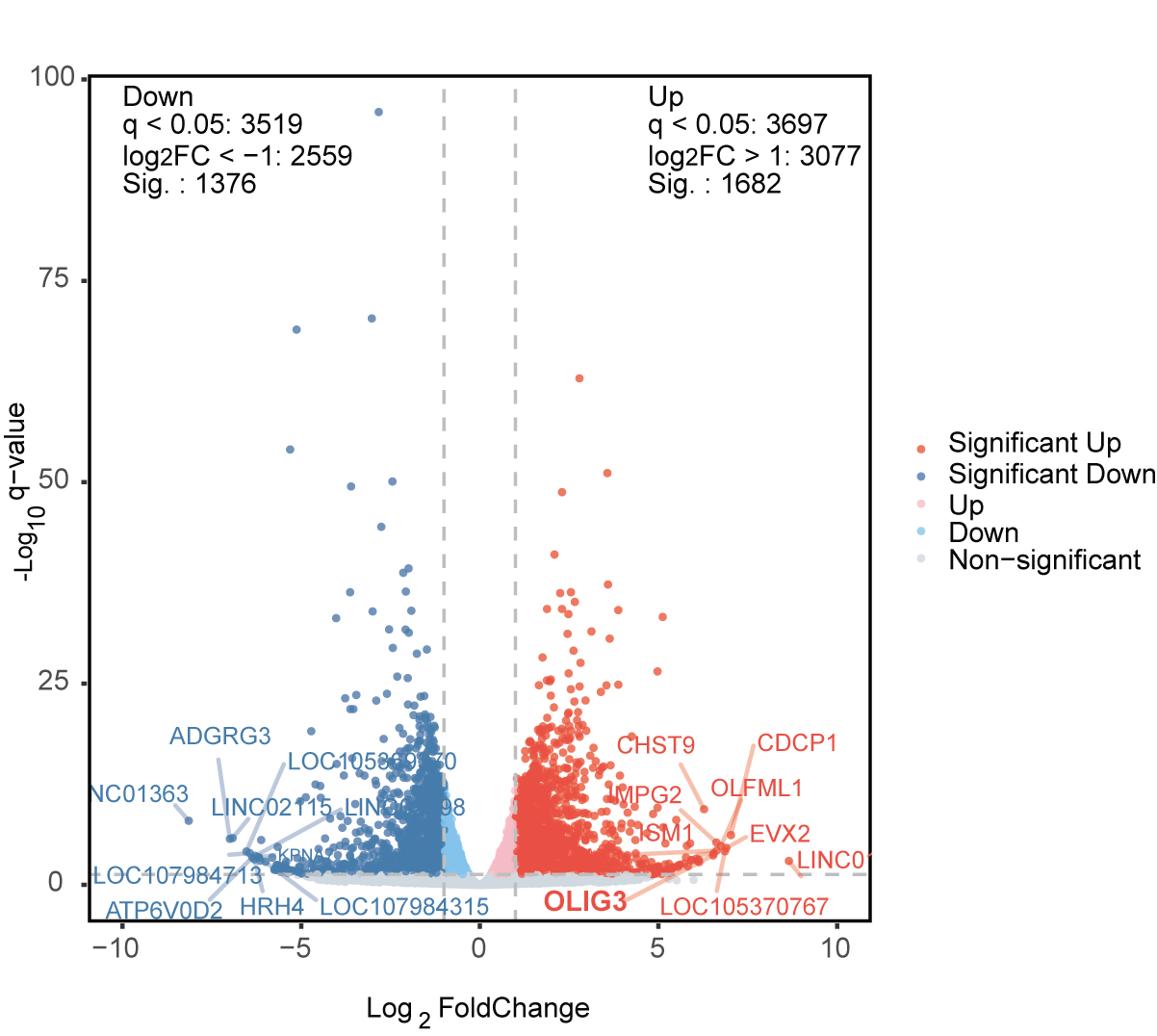

Supplement: Supplementary file 5 — Figure S4. Volcano plot of DEGs analysis between VPA treated and control hNTOs. [file CPR-58-e13737-s008.tif]

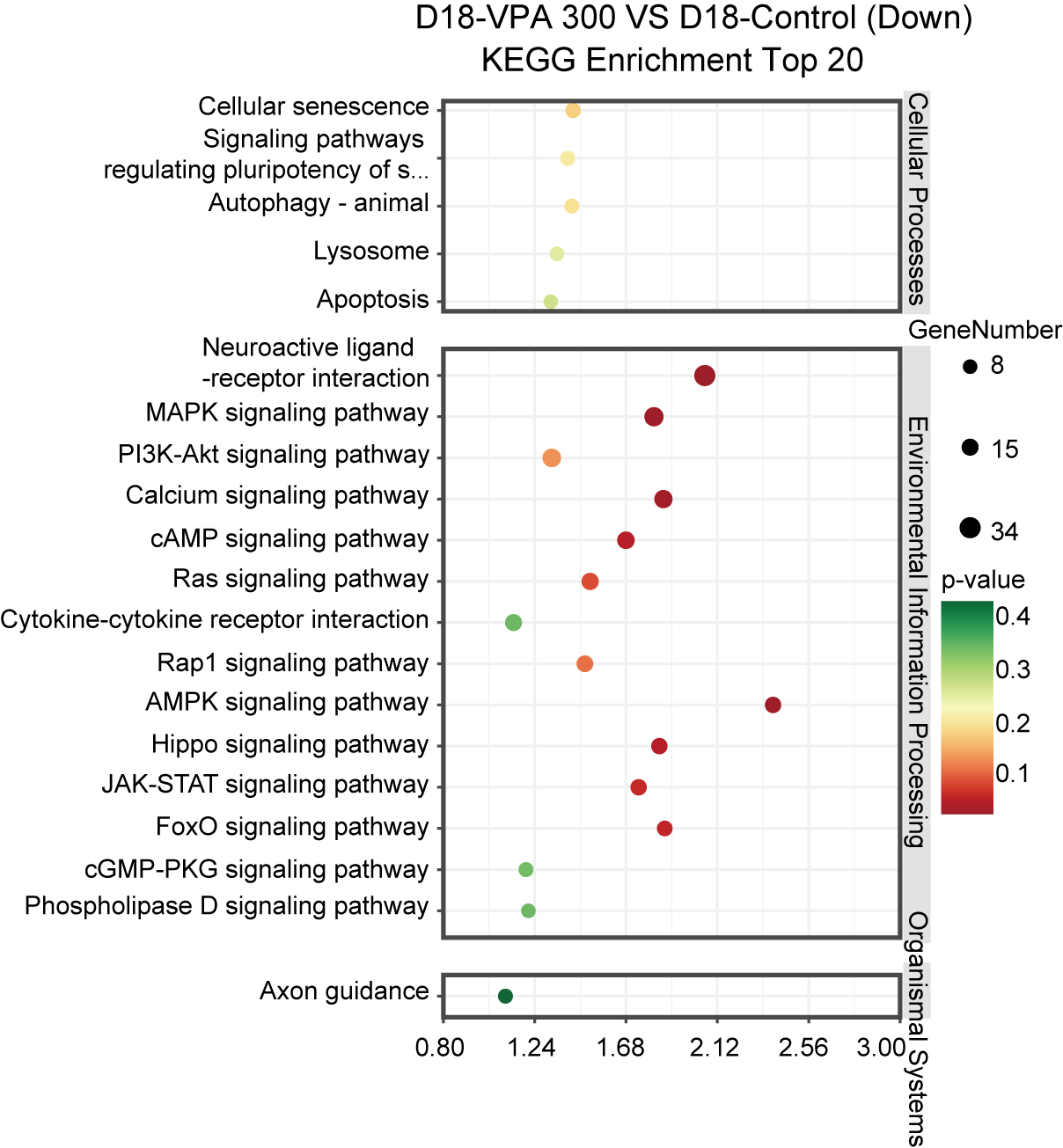

Supplement: Supplementary file 6 — Figure S5. KEGG enrichment results of down‐regulated DEGs. [file CPR-58-e13737-s003.tif]

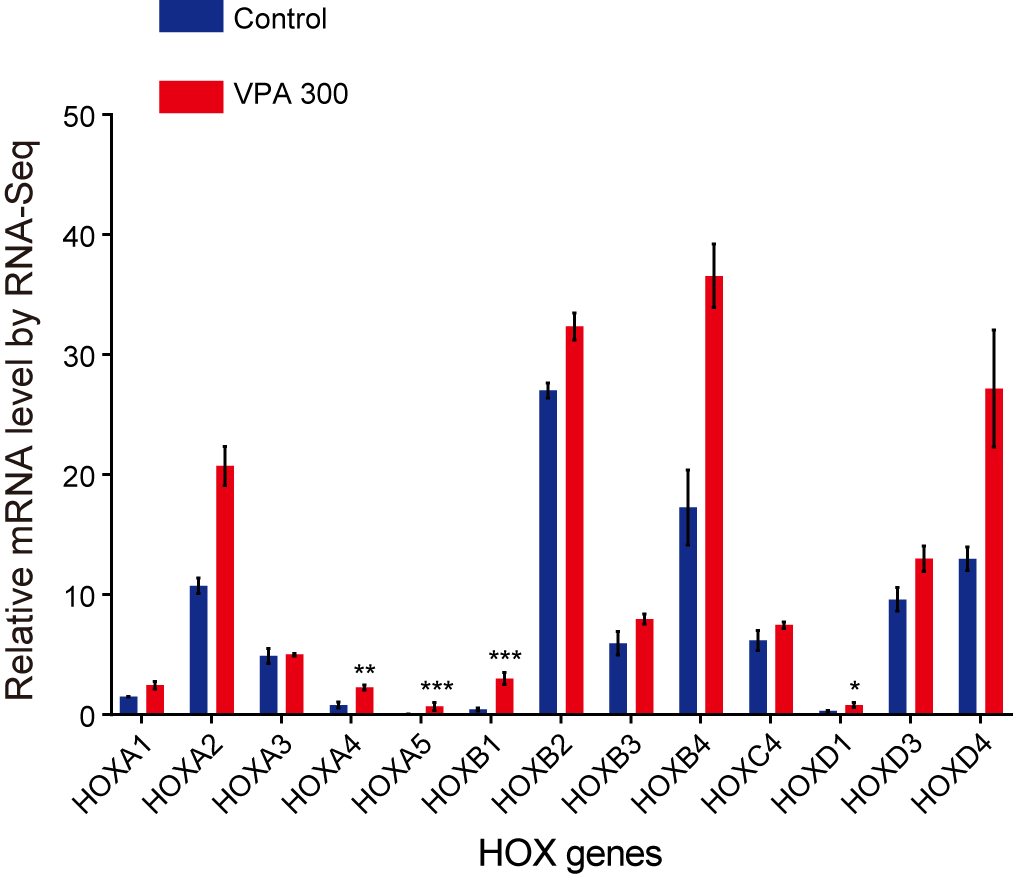

Supplement: Supplementary file 7 — Figure S6. The graph shows RNA‐seq results (normalised FPKM) of gene sets enriched in WNT signalling. [file CPR-58-e13737-s001.tif]

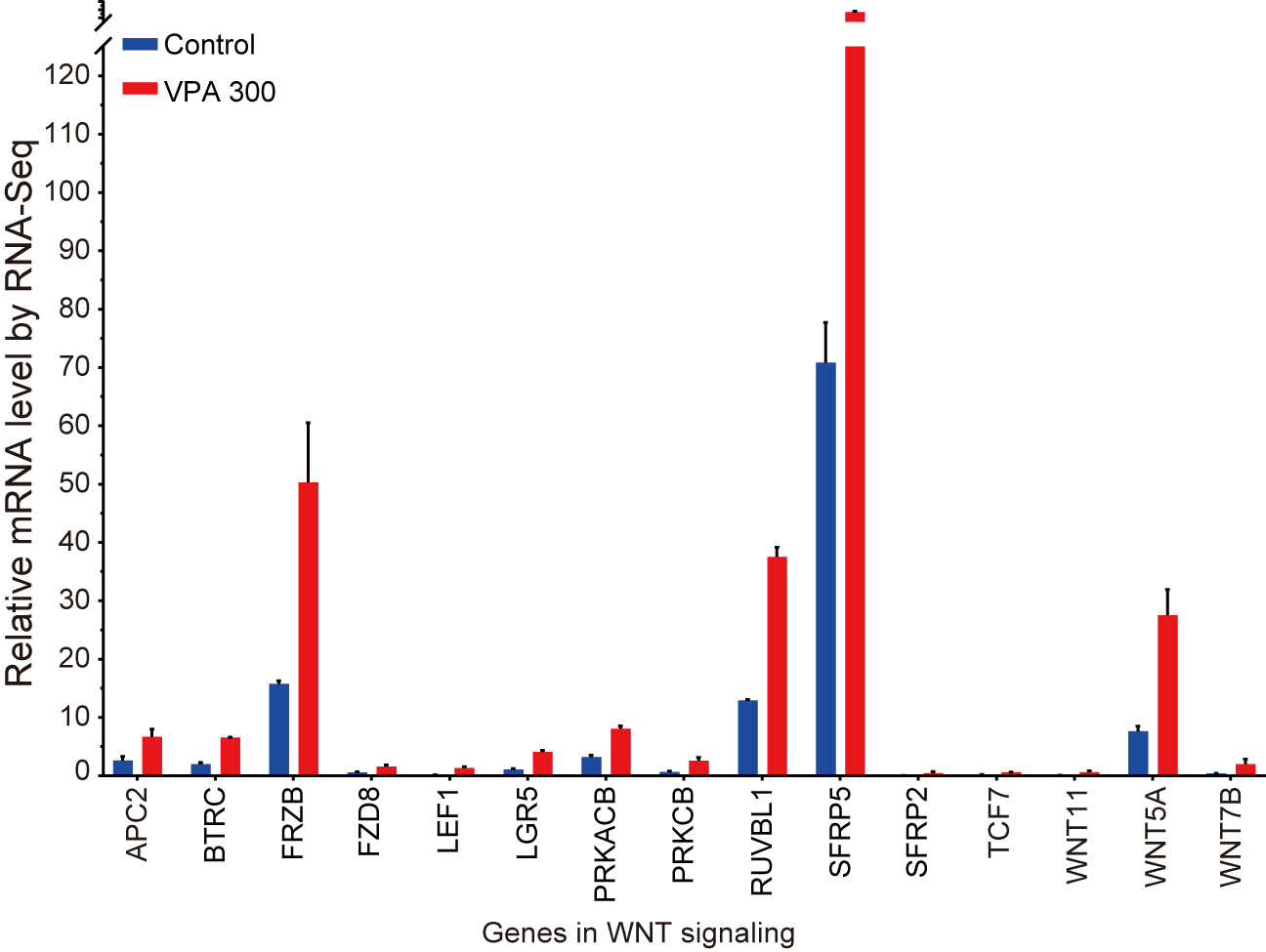

Supplement: Supplementary file 8 — Figure S7. The analysis of β‐catenin expression after treatment of hNOTs with 300 μM VPA. [file CPR-58-e13737-s010.tif]

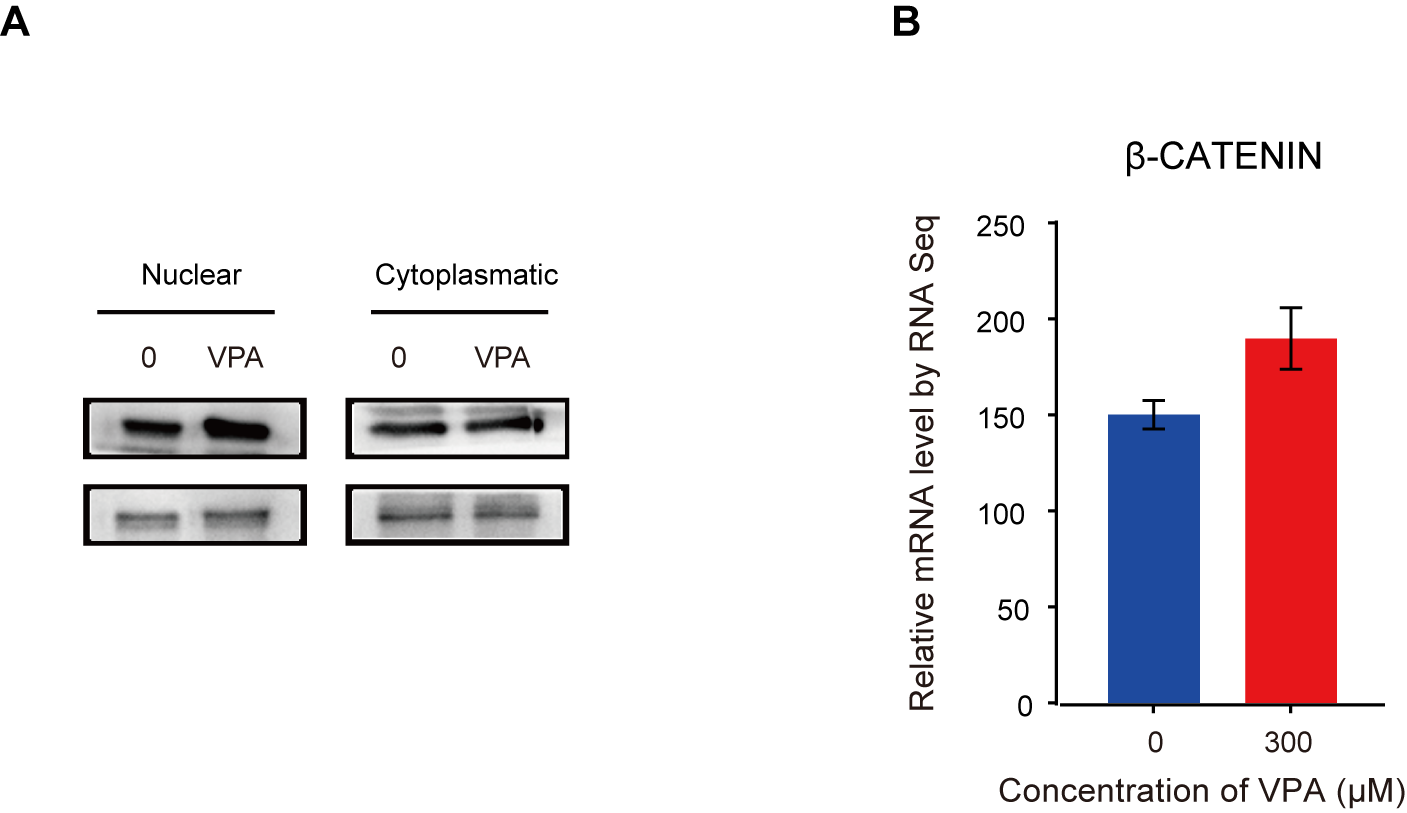

Supplement: Supplementary file 9 — Figure S8. The graph shows RNA‐seq results (normalised FPKM) of HOX (A–D) genes along AP axis. [file CPR-58-e13737-s002.tif]

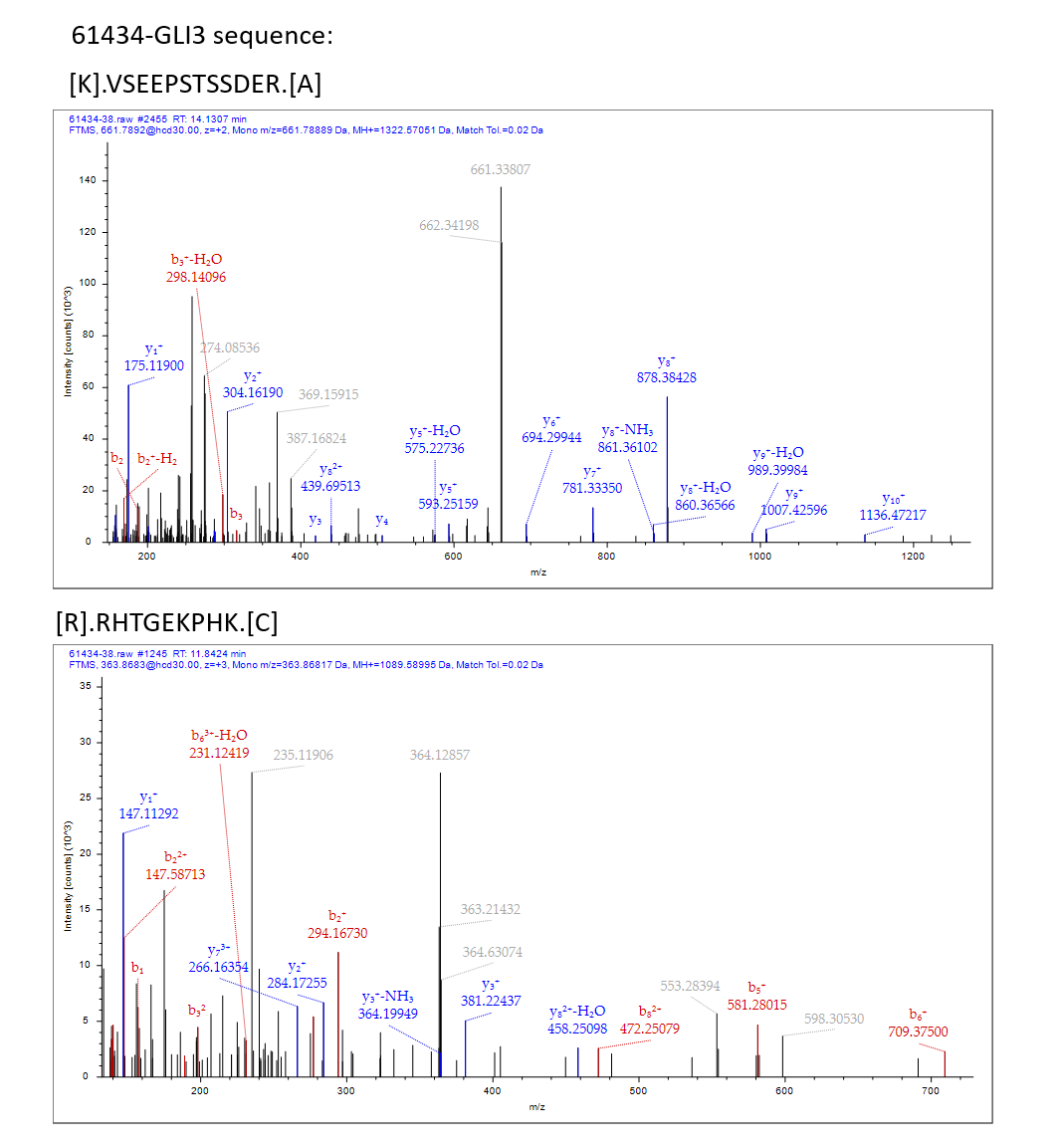

Supplement: Supplementary file 10 — Figure S9. The secondary mass spectrometry of GLI3R protein band in VPA‐treated organoids. [file CPR-58-e13737-s011.tif]

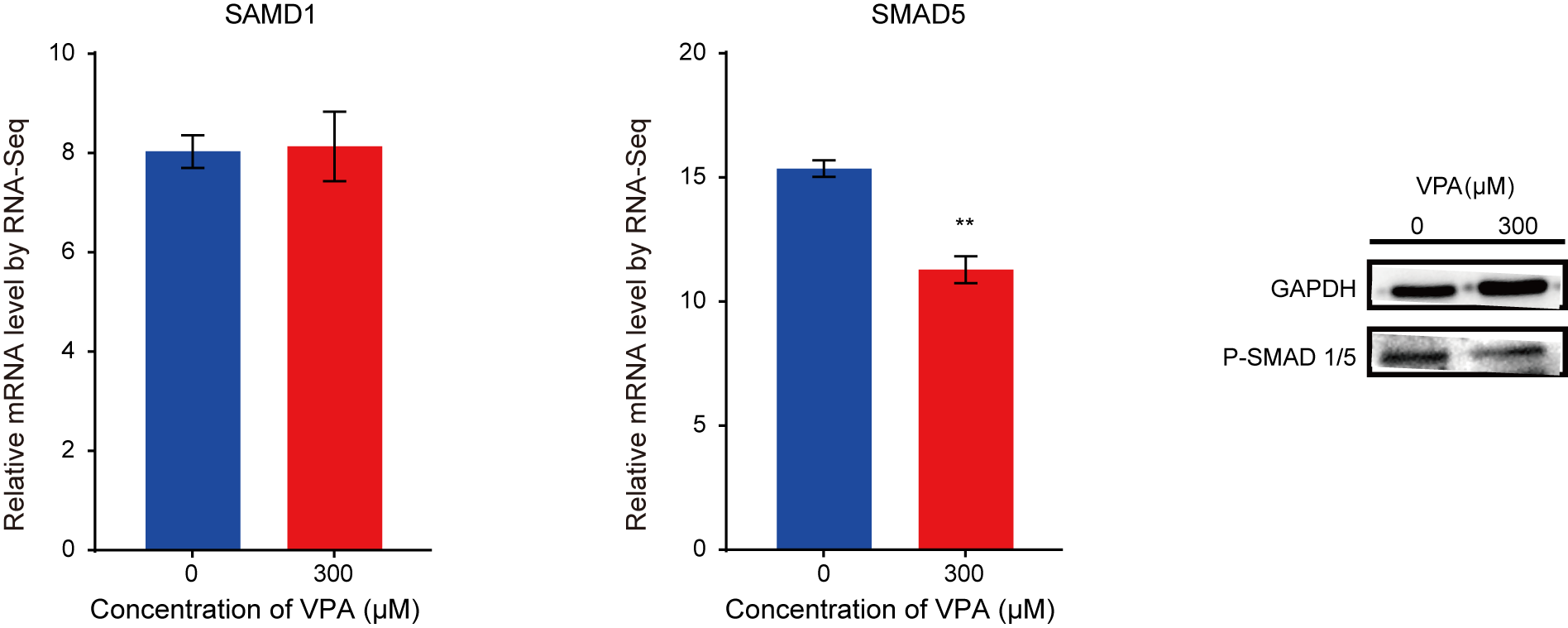

Supplement: Supplementary file 11 — Figure S10. The BMP signalling pathway was not activated by VPA. [file CPR-58-e13737-s004.tif]

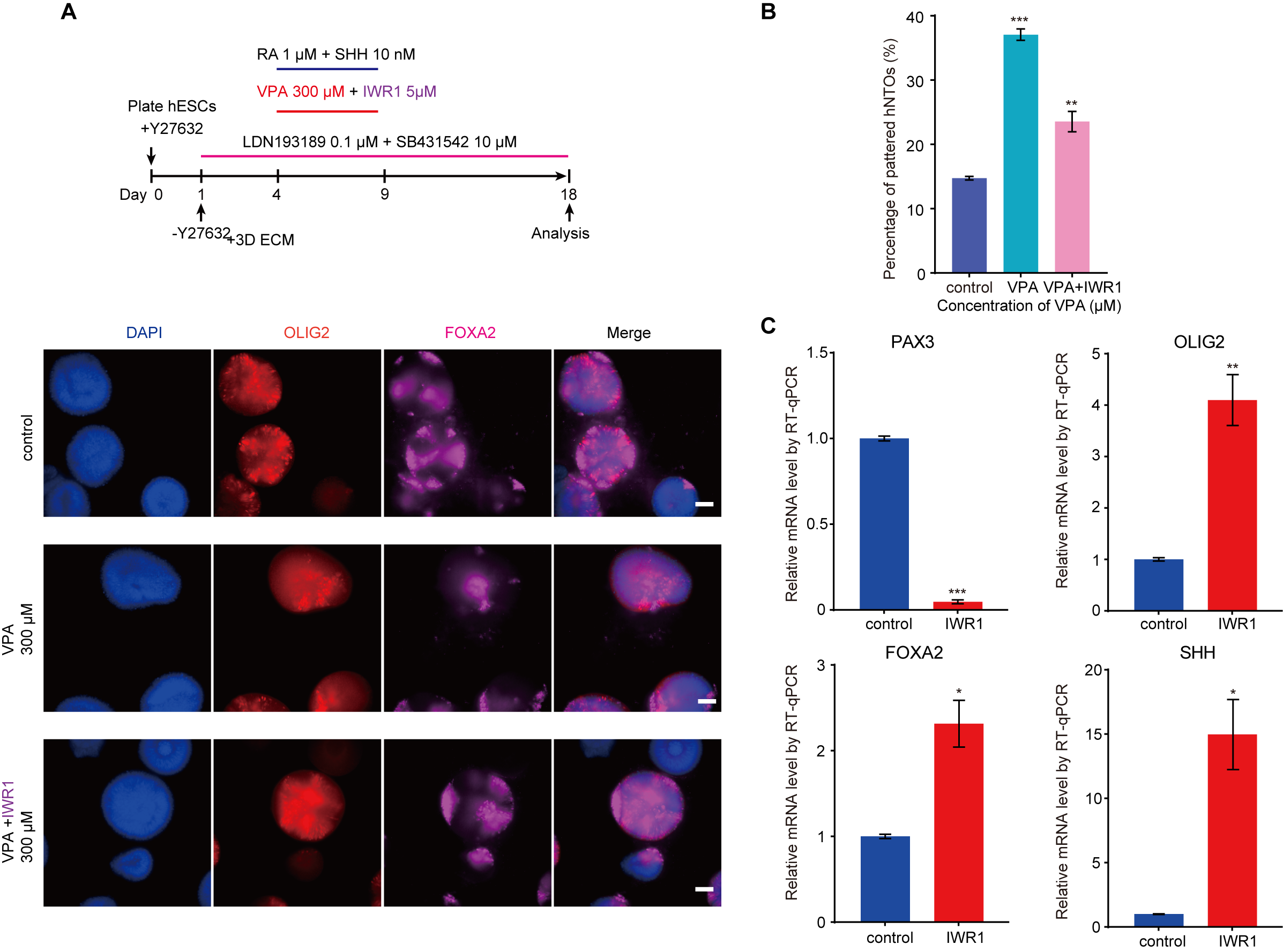

Supplement: Supplementary file 12 — Figure S11. The effect of Wnt inhibition IWR‐1 on the ventral patterning of hNTOs. [file CPR-58-e13737-s007.tif]
